# Characterization of Bimetallic Pd–Fe Nanoparticles Synthesized in *Escherichia coli*

**DOI:** 10.1021/acsabm.4c01354

**Published:** 2024-12-02

**Authors:** Ana Lucía Campaña Perilla, Jaime Gomez-Bolivar, Mohamed L. Merroun, Nadeem Joudeh, Athanasios Saragliadis, Anja Røyne, Dirk Linke, Pavlo Mikheenko

**Affiliations:** † Department of Biosciences, University of Oslo, P.O. Box 1066 Blindern, 0316 Oslo, Norway; ‡ Department of Physics, University of Oslo, P.O. Box 1048 Blindern, 0316 Oslo, Norway; § Department of Microbiology, University of Granada, Campus Fuentenueva, 18071 Granada, Spain

**Keywords:** palladium−iron nanoparticles, biogenic nanoparticles, Escherichia coli, transmission electron microscopy, catalysis, 4-nitrophenol

## Abstract

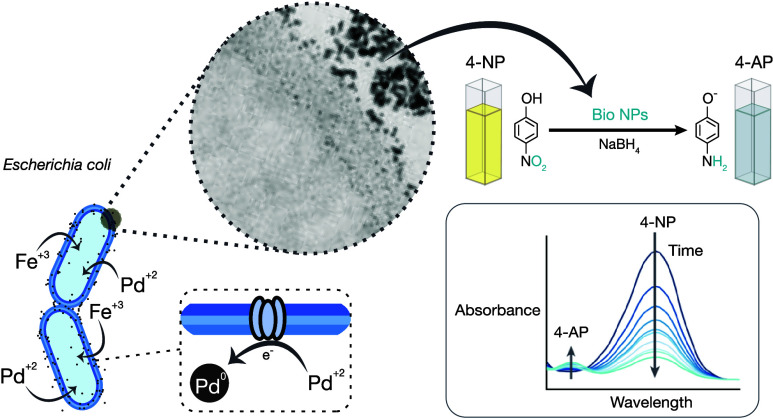

Biologically mediated nanoparticle (NP) synthesis offers a reliable and sustainable alternative route for metal NP production. Compared with conventional chemical and physical production methods that require hazardous materials and considerable energy expenditure, some microorganisms can reduce metal ions into NPs during standard metabolic processes. However, to be considered a feasible commercial option, the properties and inherent activity of bio-NPs still need to be significantly improved. In this work, we present an *Escherichia coli*-mediated synthesis method for catalytically active Pd–Fe NPs. The produced biogenic Pd–Fe NPs with varying Fe content were characterized using complementary analytical techniques to assess their size, composition, and structural properties. In addition, their catalytic performance was assessed by using standardized chemical reactions. We demonstrate that the combination of Pd with Fe leads to synergistic effects that enhance the catalytic performance of Pd NPs and make biogenic Pd–Fe NPs excellent potential substitutes for currently used catalysts. Briefly, the apparent rates for the model reaction of 4-nitrophenol reduction to 4-aminophenol catalyzed by Pd-based nanoparticles were as high as 0.1312 min^–1^ using bimetallic Pd–Fe NPs, which is far superior to the rates of monometallic Pd NPs counterparts. This study provides a feasible strategy for the synthesis of multimetallic Pd-based NPs using common microbial processes. It emphasizes the potential of biogenic Pd–Fe NPs as efficient and sustainable catalysts for hydrogenation reactions, offering an environmentally friendly synthesis for various applications, including wastewater treatment and the production of fine chemicals.

## Introduction

In order to achieve metal homeostasis, bacteria possess complex mechanisms to transport, regulate, and bind both essential and nonessential metal ions. Some bacterial species have developed mechanisms to tolerate high metal ion concentrations and even benefit from them. Metal-reducing bacteria, such as *Geobacter*, *Shewanella*, and *Desulfovibrio* species, can use various metals as alternative electron acceptors during respiration in anoxic environments.^1^ Other bacteria transform heavy metal ions into insoluble chemical compounds to overcome toxicity and to protect their integrity under stressful environmental conditions.^2,3^ The use of metals in metabolic processes as electron acceptors in anaerobic respiration is crucial in some biogeochemical processes, including metal cycling in sediments, soils, and aquatic ecosystems.^4^ The microbial reduction of certain metals often results in their further transformation into metal nanoparticles (NPs).^3,5^ These evolutionary adaptations by microorganisms can be used in circular economy concepts because of their potential use in the microbial recovery of heavy metals and the biological synthesis of useful metallic NPs. Conventional physicochemical synthesis methods for NPs often require intensive heat and/or pressure, energy-intensive instrumentation, and harmful solvents or stabilizing agents.^6−8^ The microbial synthesis of NPs potentially offers simple, cost-effective, and more environmentally friendly alternative processes.^9^

Microbes, including but not limited to bacteria, are well known for their ability to form monometallic NPs. In recent years, there has been a significant increase in research on the microbial fabrication of structured bimetallic nanoparticles, indicating a growing interest in this area.^10−13^ The synergy between metals in multimetallic nanomaterials can bring unique properties to the materials, including increased stability, enhanced catalytic activity, and unique magnetic attributes.^14^ Bimetallic NPs are expensive, tedious, and time-consuming to produce physically or chemically.^15^ In several studies on the microbial synthesis of monometallic NPs, the bacterial species *Shewanella oneidensis*,^16,17^*Desulfovibrio desulfuricans*,^18,19^ Bacillus cereus,^20,21^ and *Escherichia coli*,^22−25^ among others, have demonstrated a prominent ability to enzymatically reduce metal ions such as Pd, Pt, Au, and Ag ions to metal NPs. The biosynthesis of bimetallic NPs follows the same principles, where the cells are exposed to different metal ions in single-step or multiple-step protocols. Examples include the production of biogenic Pd–Ag, Pd–Ru, Pd–Pt, and Au–Pd NPs with improved catalytic performance and a variety of structures (core–shell, alloys, cluster-in-cluster) dependent on the synthesis methodology.^10,26−29^

A good model system for the study of bimetallic NPs is Pd-based materials. Palladium is a rare chemical element from the platinum group of metals, well known for its unique catalytic properties.^30^ The physical and chemical properties of Pd-based materials are hugely affected by their morphology and composition.^31^ The use of Pd in the form of NPs greatly increases the surface-to-volume ratio for catalysis and confers novel electronic, magnetic, and photonic properties compared to the bulk metal.^32^ In addition, the synergy between Pd and certain metals can result in the improvement of the catalytic activity. As reported by Hosseinkhani et al., the reduction of 4-nitrophenol (4-NP) by Pd–Au NPs biosynthesized by *Cupriavidus necator* cells is substantially faster than by the monometallic Pd and Au NPs.^33^ Using magnetically recoverable PdAu/Fe_3_O_4_ NPs produced by *Shewanella oneidensis* MR-1, Tuo et al.^34^ confirmed this result. In this case, magnetism of Fe_3_O_4_ was considered highly advantageous, as this feature would make catalyst recovery steps in chemical synthesis fast and efficient.^35−37^ Surprisingly, little is known about the production of Pd–Fe bimetallic NPs by microorganisms, despite their potential applications as, e.g., magnetically recoverable nanocatalysts. To the best of our knowledge, Pd–Fe NPs synthesis using *E. coli*, an organism that can be used in easily scalable production processes, has not been reported before.

An important model reaction used to evaluate the metal NP catalytic activity is the reduction of 4-NP to 4-aminophenol (4-AP) in the presence of sodium borohydride (NaBH_4_). This reaction is relevant because 4-NP is a pollutant commonly found in textile industry wastewater. It can be converted into 4-AP, a crucial intermediary for producing agrochemicals and pharmaceutical molecules.^38^ Noble metal NPs, including palladium NPs, can catalyze this reduction under mild conditions. For instance, a study by Zhao et al. demonstrated that Pd NPs supported on aluminum silicate exhibit high activity for nitroarene reductions, including 4-NP.^39^ Similarly, Limaye et al. found that biologically synthesized Pd NPs using cork extract effectively catalyze 4-NP reduction.^40^

In this paper, we report a simple bacteria-mediated synthesis of catalytically active bimetallic Pd–Fe NPs by *E. coli*. Our approach is a single-step process in which the ions of Pd and Fe are both reduced simultaneously. Different ratios of Pd/Fe were used to explore the effect of Fe concentration on the composition, structure, and catalytic and magnetic properties of the particles. By means of high-resolution transmission electron microscopy (HR-TEM) combined with energy-dispersive X-ray (EDX) spectroscopy and element mapping analysis, the composition, size distribution, and localization of the biosynthesized NPs were analyzed. In a model reaction, hydrogenation of 4-nitrophenol to 4-aminophenol, the biogenic Pd and Pd–Fe NPs were examined for their catalytic properties.

## Results and Discussion

The biological synthesis of elemental Pd NPs by bacterial cells has been described previously.^17,19,41^ Conventional biosynthesis protocols include the uptake of soluble Pd metal ions into the cells, followed by the reduction of the cations and deposition into NPs. In this work, Pd and Pd–Fe NPs with various Pd/Fe ratios were produced using *E. coli* cells by a modified version of the methodology reported by Courtney et al.^42^ (see the Methods section). The use of *E. coli* bacteria offers several advantages. Due to its rapid growth rate, *E. coli*-based production can be easily scaled up, making it suitable for industrial applications.^43^*E. coli* synthesizes NPs under mild temperatures (10–60 °C) and pressures, eliminating the need for energy-intensive processes.^41,44,45^ Additionally, bacteria naturally produce capping agents that enhance NP biocompatibility, reducing the need for synthetic stabilizers, which are often toxic or environmentally harmful.^46^ Moreover, well-characterized *E. coli* strains enable genetic engineering to introduce specific genes that express proteins or peptides to enhance, e.g., metal ion reduction. Such genetic modifications could help to further optimize biogenic synthesis, improving NP formation efficiency in future studies.^45^

Various ratios of Pd/Fe were tested in this study to find the optimal relation that generates Pd–Fe NPs with enhanced catalytic and magnetic features. Table 1 shows the nominal metal ion loading and different metal ratios of the samples.

**Table 1:** Metal Loading of the Bacterial Samples^a^

	nominal % w/w		actual % w/w		
	Pd	Fe	Pd	Fe	total metal loading (% w/w)
*E. coli*-Pd	10%	0	8.9%	0	8.9%
*E. coli*-Fe	0	5.0%	0	5.0%	5.0%
*E. coli*-Pd/Fe_0.5_	10%	2.5%	9.6%	2.5%	12.1%
*E. coli*-Pd/Fe_1_	10%	5.0%	9.7%	4.8%	14.5%
*E. coli*-Pd/Fe_2_	10%	10%	9.5%	7.7%	17.2%
*E. coli* HK-Pd	10%	0	3.9%	0	3.9%

a Nominal and actual metal loading of *E. coli* after 2 h of exposure to metal solutions (% metal weight/dry cell weight). Nominal loading was calculated based on the measured uptake, while actual loading was determined by atomic absorption spectroscopy (AAS).

### Uptake of Pd(II) and Fe(III)

The synthesis of Pd and Pd–Fe NPs started with exposure of the bacteria to a medium that included Na_2_PdCl_4_ and FeCl_3_. After the incubation of the *E. coli* cells with the respective concentrations of Pd and Fe ions and subsequent centrifugation, the cell pellet changes color from ivory to dark yellow, indicating bacterial metal uptake. The uptake measured by the tin(II) chloride assay showed that the removal of Pd(II) ions from the solutions by the *E. coli* cells is >92% within 2 h, while for the control samples with heat-killed cells, it is only around 20% (Supporting Figure S1). A similar behavior was observed by Deplanche et al. in the biological synthesis of monometallic Pd NPs.^47^ The limited uptake by the heat-killed control samples is likely attributed to the chemical adsorption of metal ions by the membranes of dead bacteria. At the same time, this suggests that the removal of Pd(II) by living bacterial cells is mediated not only by physicochemical interactions but also mainly by active metabolic processes.

The reduction of metal ions and the formation of NPs in our workflow is initiated and supported by the addition of sodium formate, which plays the role of external electron donor for the reduction of the metals.^48^ After 1 h of incubation with sodium formate, the color of the cell suspension turns to black due to the reduction of the metal ions and the formation of NPs.^28^ In contrast, the color of the heat-killed cell suspension remains yellow, confirming that the reduction and NP formation are promoted only by metabolically active, live bacteria. It is worth mentioning, though, that the reduction of Pd and Pd–Fe may eventually happen in the heat-killed bacterial samples or samples without external electron donors after longer periods of incubation (>24 h).^48^ This slow reduction is presumably due to passive interactions with the biological material and spontaneous photochemical reactions.

**Figure 1:**
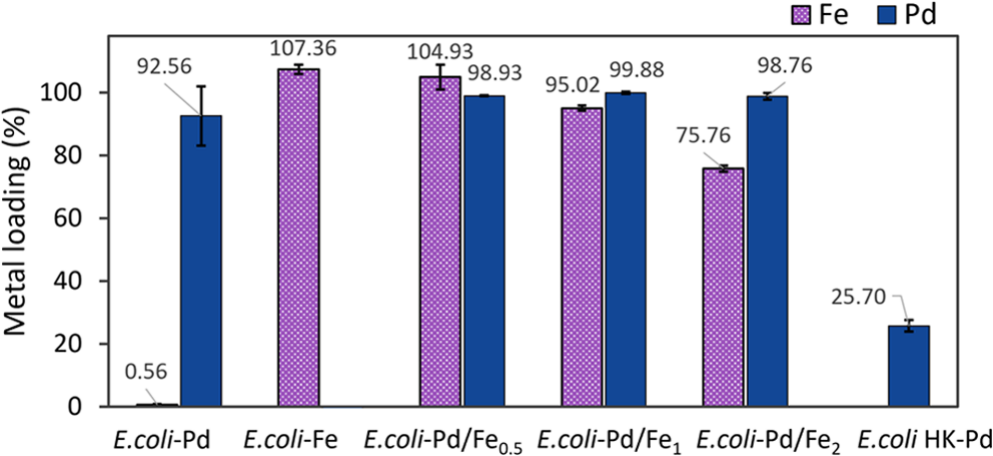
Pd and Fe ion uptake. *E. coli* cells were incubated for 2 h with different ion concentrations (Pd/Fe ratios 1:0.5, 1:1, and 1:2). The mass ratio is based on AAS measurements.

The metal uptake of Pd and Pd–Fe samples was determined by AAS and is presented in Figure 1. Although the complete removal of Pd(II) has been reported to occur after ∼30 min of incubation,^18,28^ the tin(II) chloride assay results (Supporting Figure S1) suggest that an almost complete removal of Pd(II) is achieved only within 2 h of exposure for bacteria exposed only to Pd. Therefore, the uptake of metal ions was performed with cells incubated for 2 h. At this time point, the average Pd(II) uptake for the monometallic *E. coli*-Pd sample is 92.5 ± 9.4%, while for the bimetallic samples, the uptake is higher than 98% (Figure 1). In the case of Fe, the removal of Fe from the medium decreases with an increase in its concentration. The *E. coli*-Pd/Fe_0.5_, *E. coli*-Pd/Fe_1_, and *E. coli*-Pd/Fe_2_ samples recovered 100 ± 3.4, 95.0 ± 0.84, and 75.7 ± 1.0% of the Fe added to the medium, respectively, and the cells incubated with Fe only showed an apparent uptake slightly higher than the total amount of the element added. This is because Fe is a nutrient essential for cell survival, and the medium in which the bacteria cells are grown already contains traces of Fe necessary for their metabolic process. The values of Fe uptake exceeding 100% thus may include additional Fe extracted from the medium. The decrease of the recovered amount of Fe in the Pd–Fe samples is proportional to the initial metal concentration, suggesting a saturation of all of the available sites for metal absorption in the biomass. Bacteria possess complex regulatory pathways for Fe homeostasis that regulate the Fe ion uptake and its sequestration after reaching a certain intracellular concentration.^49^

Therefore, the actual metal content in each sample differs slightly from the nominal one, as shown in Table 1. In further experiments, the actual values were determined by subtracting the nonrecovered metal from the initially added metal mass. These values were used to normalize downstream experiments (e.g., catalyst performance measurements. See below).

### General Properties of the Biosynthetic Pd and Pd–Fe NPs

#### Functional Groups Associated with Pd and Pd–Fe NP Synthesis

**Figure 2:**
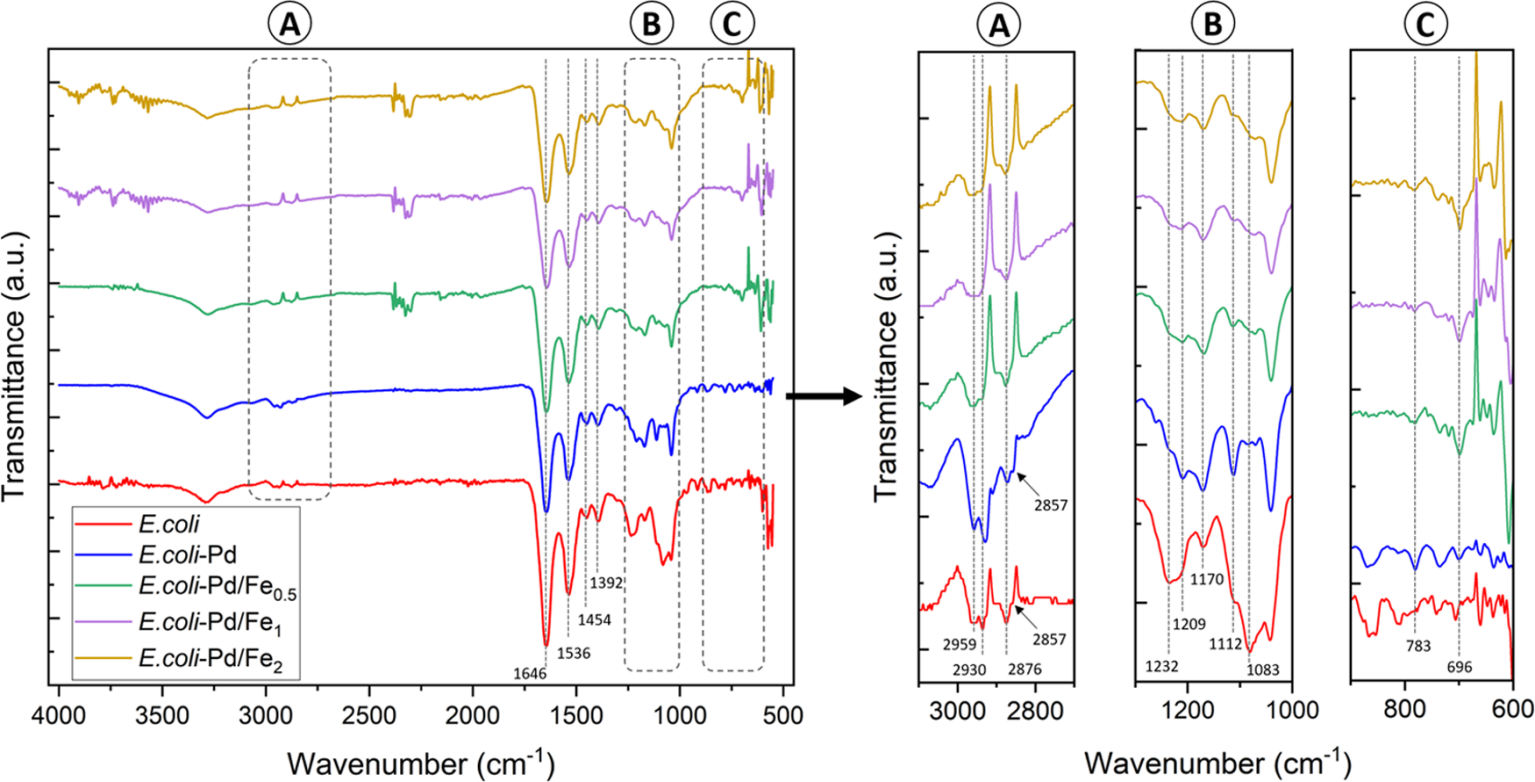
Infrared spectra of the samples. Left: full FT-IR spectra of *E. coli* samples with biosynthesized Pd NPs and Pd–Fe NPs in ratios 1:0.5, 1:1, and 1:2. Right: a closer view at the regions between (A) 3100–2700 cm^–1^, (B) 1300–1000 cm^–1^, and (C) 900–600 cm^–1^.

Fourier transform infrared (FT-IR) spectrometry analysis was conducted to determine the relevant functional groups in bacterial macromolecules associated with biosynthesized Pd and Pd–Fe NPs. Figure 2 displays the spectra of the NP samples, and Table 2 summarizes the most relevant functional groups related to bacterial interaction with Pd and Fe NPs. Strong bands in the range 3500–3200 cm^–1^ are associated with the stretching of the N–H bond of amino groups and the O–H stretching vibration.^50^ The spectrum of untreated bacterial biomass presents a broad peak at 3280 cm^–1^, and similar broad peaks are found in samples loaded with Pd and Fe, which reflect the properties of bacterial polysaccharides and proteins.^51^ The peaks around 3000–2800 cm^–1^ are ascribed to C–H bonds stretching in fatty acids.^50,52^ The asymmetric stretching of the −CH_3_ groups combined with those of the −CH_2_ groups were found at 2959 and 2876 cm^–1^, while the symmetric stretching is seen at 2930 and 2857 cm^–1^, in *E. coli* biomass. Upon loading with Pd and Fe, these peaks decrease in intensity, showing variation in the band area or a shift in frequency. Cagnasso et al.^53^ suggested that this may be indicative of the reorganization of fatty acid chain structure in phosphatidic acid at the bacterial membranes interacting with iron oxides such as hematite. However, several studies have shown alteration in lipids and fatty acids concentration, structure, and composition due to heavy metal stress following exposure to Cd, Pb, Ni, and Cr.^54,55^ The decrease and upward shift of the bands observed in the *E. coli*-Pd sample may therefore demonstrate a similar alteration in lipids or a conformational disorder in the fatty acid chains due to Pd and Fe toxicity.

**Table 2:** Functional Groups Relevant to the Observed FT-IR Spectra from Figure 2

wavenumber range (cm^–1^)	wavenumber (cm^–1^)	functional group		description	modification	refs
3500–3200	3280	N–H, O–H	stretching	polysaccharides and proteins		(51)

3000–2800	2959	–CH_3_	asymmetric stretching	fatty acids	decrease in intensity after Pd and Fe exposure	(50,52)
	2930	–CH_3_	symmetric stretching			
	2876	–CH_2_	asymmetric stretching			
	2857	–CH_2_	symmetric stretching			

1800–1300	1646	C=O, C–N	stretching	peptide bonds in proteins		(55)
	1536	C–N, N–H	stretching, bending			(55)
	1454	C–H	bending	lipids and fatty acids		(54)
	1392	COO–	symmetric stretching			(54)
	1307	C–N, N–H	stretching, bending	peptide bonds		(56)

1300–1000 fingerprint region	1232	P=O	asymmetric stretching	nucleic acids and phospholipids	decrease in intensity after Pd and Fe exposure	(57,58)
	1209	P=O			new band after Pd and Pd–Fe exposure	this work
	1170	CO–O–C	asymmetric stretching			(52,54)
	1112	P=O			new band after Pd and Pd–Fe exposure	this work
	1083	P=O	symmetric stretching		decrease in intensity after Pd and Fe exposure	(52,53)
	1040	P–O	stretching			(53,57)

1000–650 fingerprint region	783	Pd–O, Pd(0)	stretching		new band after Pd and Pd–Fe exposure	(59)
	696	Fe–O	stretching		new band after Fe exposure	(60)

The strong bands centered at 1646 and 1536 cm^–1^, found in all of the samples, were attributed to stretching of the C=O group in amide I and due to bending of N–H bonds and C–N stretching in amide II, respectively, which are all characteristic of the absorption spectrum of peptide bonds in proteins.^55^ The peaks located at 1454 cm^–1^ were assigned to C–H bending and at 1392 cm^–1^ to the symmetric stretching of COO^–^ groups in lipids and fatty acids.^54^ The amide III band also gives a weak peak at 1307 cm^–1.^^56^

The fingerprint region found in the range between 1300 and 500 cm^–1^ is a complex pattern of peaks unique to each sample. The unloaded control *E. coli* cells show strong bands at 1232 cm^–1^, 1170, 1083, and 1040 cm^–1^ that were assigned to asymmetric P=O stretching vibrations in nucleic acids and phospholipids,^52,57,58^ CO–O–C antisymmetric stretching of phospholipids,^52,54^ the complementary P=O symmetric stretching,^52,53^ and P–O bond stretching,^53,57^ respectively. As a result of exposure to both metals, the bands at 1232 and 1083 cm^–1^ related to P=O bonds diminished, while additional peaks appeared at 1209 and 1112 cm^–1^, particularly strongly in the spectra of *E. coli* exposed to Pd only. It was previously suggested that certain elements, such as phosphorus, might play a role in the deposition of palladium due to their colocalization in the cells.^10,41^

The vast majority of peaks in the range of 1000–650 cm^–1^ subside with the addition of Fe, with the exception of the peak at 783 cm^–1^ associated with Pd–O and Pd(0).^59^ The peak at 696 cm^–1^ on the Pd–Fe NPs samples was attributed to the stretching vibration mode of Fe–O bonds.^60^ The observed variations of the characteristic bands of the bacterial biomass, such as shift or decrease in band intensity, along with the appearance of new peaks, indicate an interaction of Pd and Fe primarily with P=O, −CH_2_, and −CH_3_ functional groups (Table 2). Hence, these modifications suggest that it is highly plausible that these groups contribute to bacterial metal uptake and further nanoparticle growth.^58^

#### Structural Features of Microbial Pd and Pd–Fe NPs

**Figure 3:**
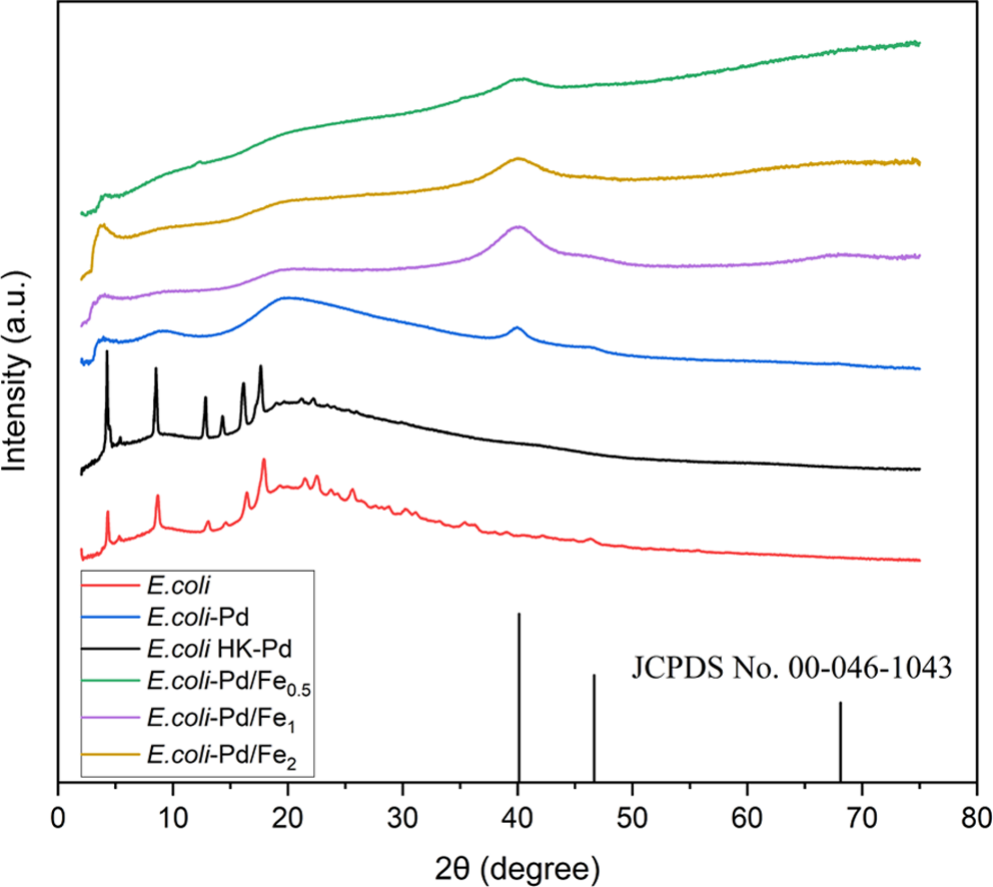
XRD patterns of the samples compared to the JCPDS card No. 046–1043 peak positions indexed to face-centered cubic (FCC) Pd standard.

X-ray diffractometry (XRD) was used to determine the crystalline structure of biosynthesized Pd and Pd–Fe NPs (Figure 3). A major peak at 40.1° and minor peaks at 46.4 and 68.4° are seen in the samples loaded with Pd. The comparison of the peaks with the standard JCPDS card No. 046–1043 peak positions indicates the presence of the {111}, {200}, and {220} lattice planes of face-centered cubic (FCC) palladium in the material. Unfortunately, the background signal attributed to the bacterial biomass and other amorphous structures in the samples obstructs the calculation of the average crystal size of the NPs. The peaks at 2θ = 4.2, 5.3, 8.6, 13.0, 14.6, 16.4, 17.9, 21.5, and 22.6° in the diffraction curves of the control samples with bacterial biomass and heat-killed bacteria loaded with Pd are attributed to crystalline bacterial surface cell proteins.^61,62^ The decrease in intensity or disappearance of biomass-related peaks in the NPs samples suggests a degradation or reorganization of the crystal structures in the bacterial proteins due to the interaction with the heavy metals. The patterns did not present other distinctive peaks that could be related to Fe or Fe oxide lattice planes (Supporting Figure S2). However, the resemblance of curves in the samples loaded with Fe to the data presented by Yadav et al.^63^ suggest the presence of amorphous Fe structures. Ultimately, the XRD patterns show the formation of FCC Pd and indicate the disruption of the bacterial ordered molecules due to exposure to Pd and Fe.

#### Biogenic Pd and Pd–Fe NP Composition and Thermal Stability

**Figure 4:**
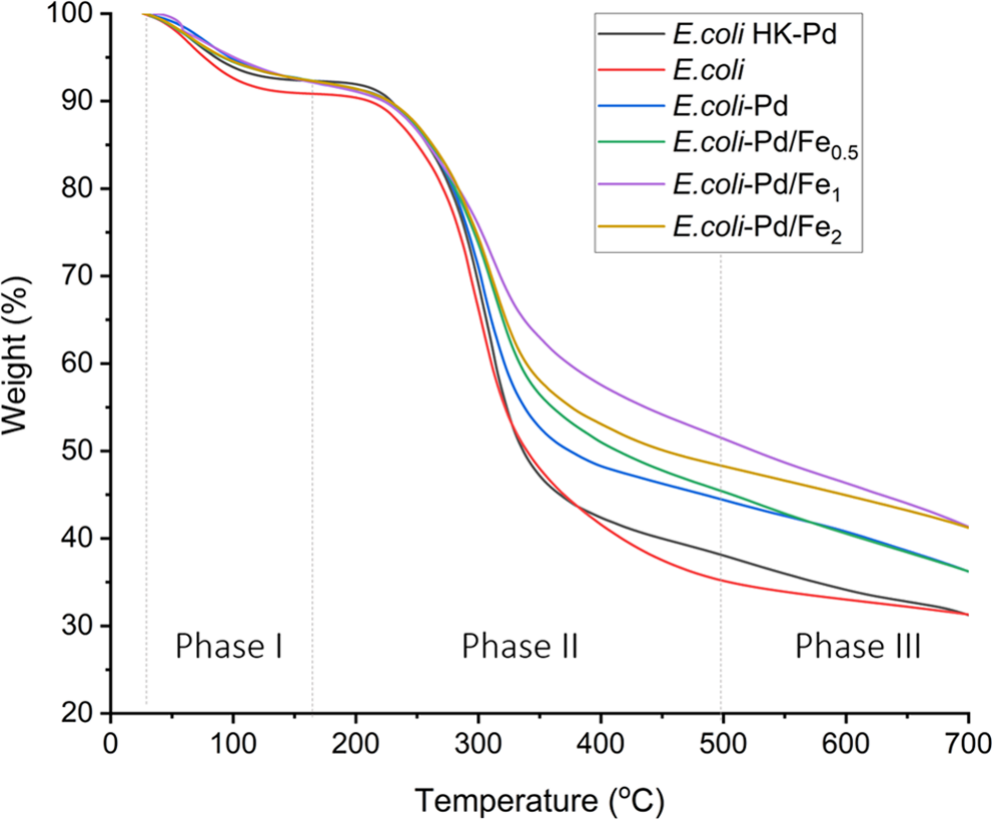
TGA curves of the samples.

Thermal gravimetric analysis (TGA) measures changes in mass as a function of temperature, helping to understand thermal stability and decomposition processes in the samples.^64^ The correspondence between the TGA curves (Figure 4) and the peaks in the differential thermogravimetry (DTG) curves (derivative weight loss) allows us to distinguish three phases of thermal decomposition (Supporting Figure S3). The initial phase corresponding to weight loss at temperatures below 170 °C is attributed to water evaporation and other solvent losses.^65−67^ This is followed by the degradation of simple organic molecules at temperatures from 170 to 500 °C. Finally, at temperatures higher than 500 °C, the decomposition of large macromolecules and the slow breakage of inorganic materials such as minerals, oxides, and carbon black takes place, as suggested in ref (68).

The TGA study of the Pd and Pd–Fe NPs shown in Figure 4 exhibits the thermal stability of the NPs and a weight loss related to biomass during a heating ramp from room temperature to 700 °C. In the first phase, the weight decreases by 10% despite the freeze-drying process. Residual water remains in the samples, which is true for all of the samples, including the controls of *E. coli* biomass and heat-killed cells loaded with Pd. This was followed by *E. coli* biomass degradation in the second phase. The changes in this interval have been associated with the degradation of cell wall components, carbohydrates, and the breakage of polysaccharides.^69^ A reduced weight loss or degradation here could indicate a smaller concentration of simple organic molecules in the samples. This is in agreement with our FT-IR results (Table 2), showing that cells loaded with Pd and Fe have lower amounts of lipids or fatty acid chains due to the ion sequestration and metal toxicity.

In the last phase, due to its elevated evaporation temperature (>1500 °C), the remaining material is likely composed mainly of biochar and inorganic elements, such as Pd and Fe. The metal loading in the cells, calculated from the TGA results, was 6.7, 6.8, 11.8, and 11.7% for *E. coli*-Pd, *E. coli*-Pd/Fe_0.5_, *E. coli*-Pd/Fe_1_, and *E. coli*-Pd/Fe_2_, respectively. Compared to the AAS results (Table 1), TGA suggests slightly lower metal uptake by all samples. It is worth noting that the final weights of the control samples, *E. coli* biomass (31.3%), and the heat-killed cells loaded with Pd trace (31.2%) samples are comparable. Considering that the *E. coli* HK-Pd sample takes up 20% of Pd ions in the medium (see the tin(II) chloride assay above and Supporting Figure S1), the final weight percentage of Pd in the sample should be around 3.9% w/w (Table 1, % metal weight/dry cell weight). This weight percentage is too small to be visualized by this method, which explains the differences in metal uptake observed between these results and the AAS study.

#### Examination of Pd and Pd–Fe NPs by Electron Microscopy and EDX

**Figure 5:**
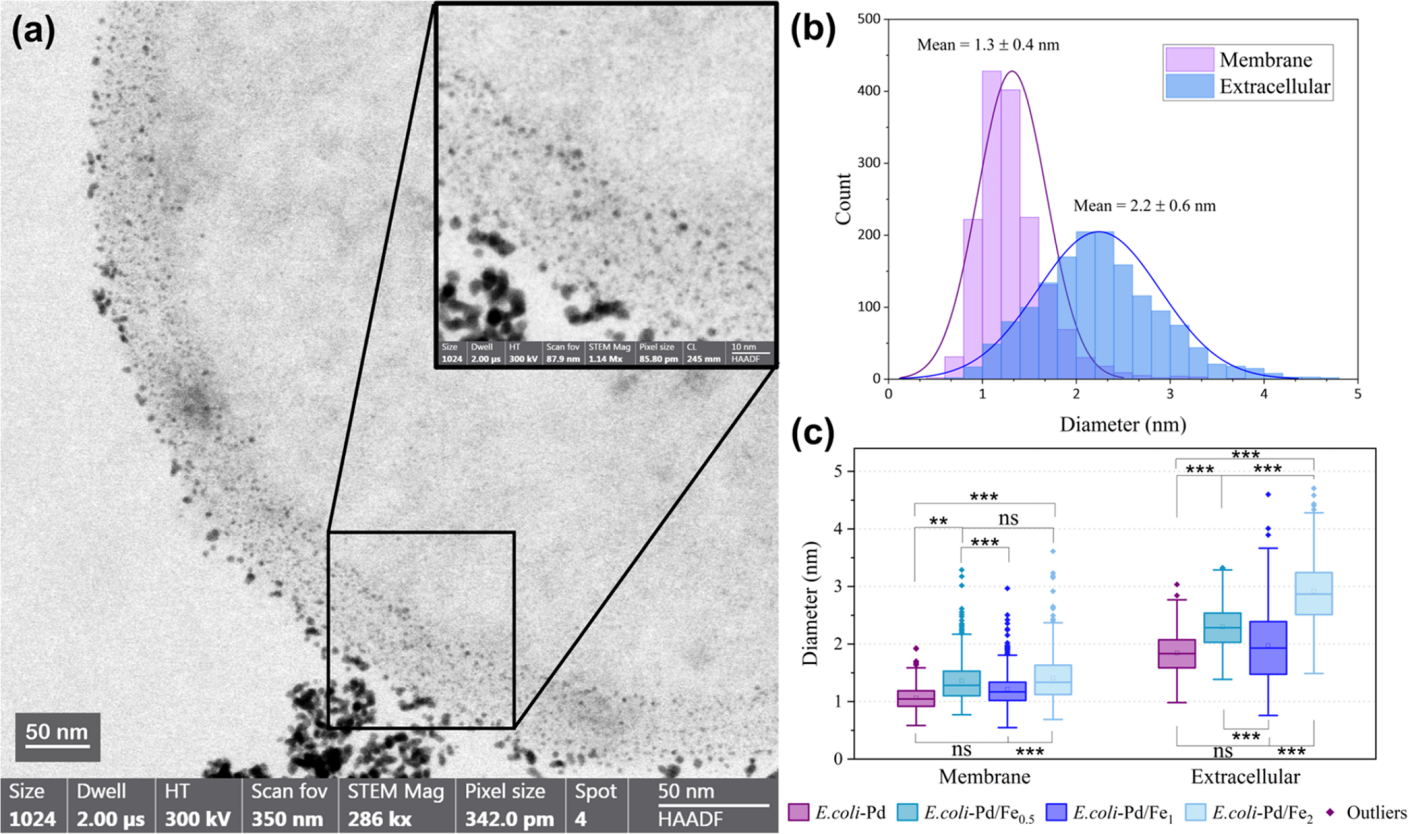
(a) High-angle annular dark-field scanning electron microscope (HAADF-STEM) micrograph of the *E. coli*-Pd/Fe_1_ sample (inverted contrast) and nanoparticle size distribution by (b) localization and (c) composition of Pd and Pd–Fe NPs (for HAADF-STEM images of other Pd and Pd–Fe samples, see Supporting Figure S5). ns = not significant.

The examination of *E. coli* cross sections by electron microscopy confirms the formation of Pd and Pd–Fe NPs in the samples. The deposits of round nanoparticles are predominantly extracellular but can also be found on the cell surface or bound to the different layers of the bacterial membranes (Figure 5a). A similar pattern of NP deposition was described by Courtney et al.^42^ for Pd NPs on *E. coli* MC4100 also using sodium formate as an electron donor. Their study suggests that intracellular deposition of Pd NPs may occur due to the involvement of membrane-bound cytoplasmic facing hydrogenase 3. Accordingly, various NPs located in the layers of the membrane and in the cytoplasm can be expected. In our samples, small and well-defined NPs were found at the bacteria membrane, and just a few cases of well-distributed NPs in the cell cytoplasm were visible. The use of H_2_ as a reduction agent by other production protocols resulted in NP deposits in the cytoplasm and periplasm, in addition to large agglomerations of surface-bound NPs.^26,42^ This suggests that the NP properties can be influenced by the choice of electron donor,^18^ including their size, morphology, and localization. However, the association of NPs localization with electron donors is still not clear. For the controls with heat-killed and unloaded cells, scanning transmission electron microscopy (STEM) showed no electron-opaque deposits extracellularly and no evidence of NPs on the bacterial cell surface or intracellularly (Supporting Figure S4). Therefore, we can conclude that the synthesis of the Pd and Pd–Fe NPs was mostly performed through a biological pathway and was not due to a spontaneous chemical reaction. In other words, the metabolic processes of living bacteria are vital for the efficient production of NPs using *E. coli*.

A size distribution analysis of the NPs was carried out using high-resolution transmission electron microscopy (HR-TEM) imaging, and the data were acquired manually with the software ImageJ. At high magnifications, the images revealed that NP size differences depend on the region of formation. The measured average particle size of the NPs bound to the cell membrane is 1.3 ± 0.4 nm, in contrast to 2.2 ± 0.6 nm for the extracellular NPs (Figure 5b). A Kruskal–Wallis *H* test showed that there was a statistically significant difference between the means of the NP size of both populations with a significance level of 0.05 (*P* = 0.0001). Therefore, the localization of NP nucleation sites has relevance to the final size distribution. At the membrane or intracellularly, the mechanisms of bacterial-metal interaction may result in the assembly of biocapping agents such as proteins and polysaccharides, which encapsulate the Pd and Fe metal ions, preventing their aggregation and promoting the formation of smaller NPs.^46^ The NPs formed in the cell membrane have a more homogeneous particle size distribution, as seen from their lower dispersity index (24.88%) compared to that (31.94%) for the extracellular populations. The addition of Fe also affects particle size (Figure 5c). The concentration of Fe seems to be correlated with the mean particle size for both extracellular and intracellular nanoparticles.

**Figure 6:**
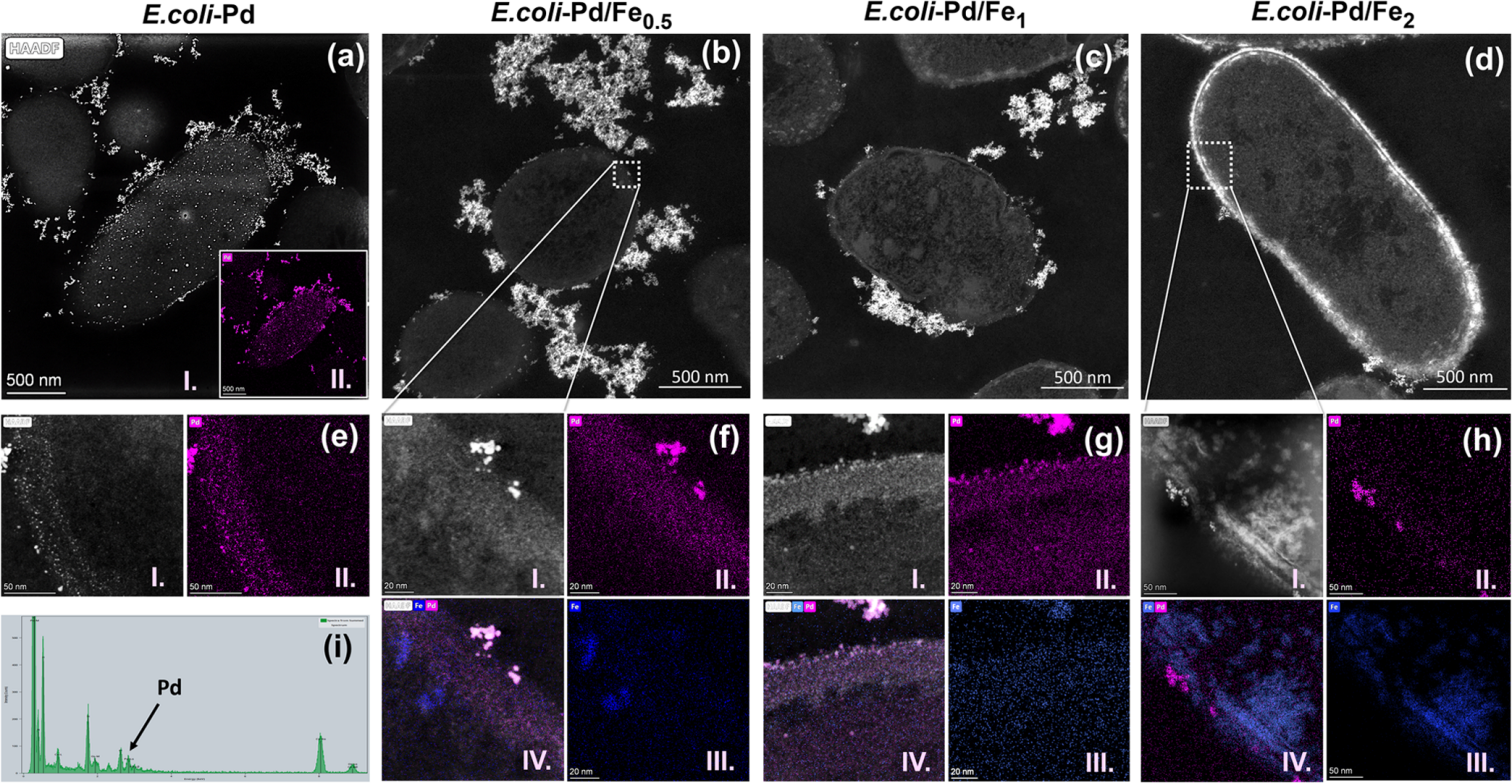
HAADF-STEM images of *E. coli* K-12 bacteria and magnified view of cell surface loaded with (a, e) Pd only and Pd/Fe (b, f) 1:0.5, (c, g) 1:1, and (d, h) 1:2 solutions. The metal distribution in the samples was studied with the backscattered electron images (I), the elemental maps for Pd (II), Fe (III), and the colocalization maps (IV) on extracellular and intracellular nanoparticles. EDX spectra analysis confirmed the presence of Pd (i) and Pd–Fe (Supporting Figure S6) in the cell surface NPs. Scale bar in panels (e, h) is 50 and 20 nm in panels (f, g).

**Figure 7:**
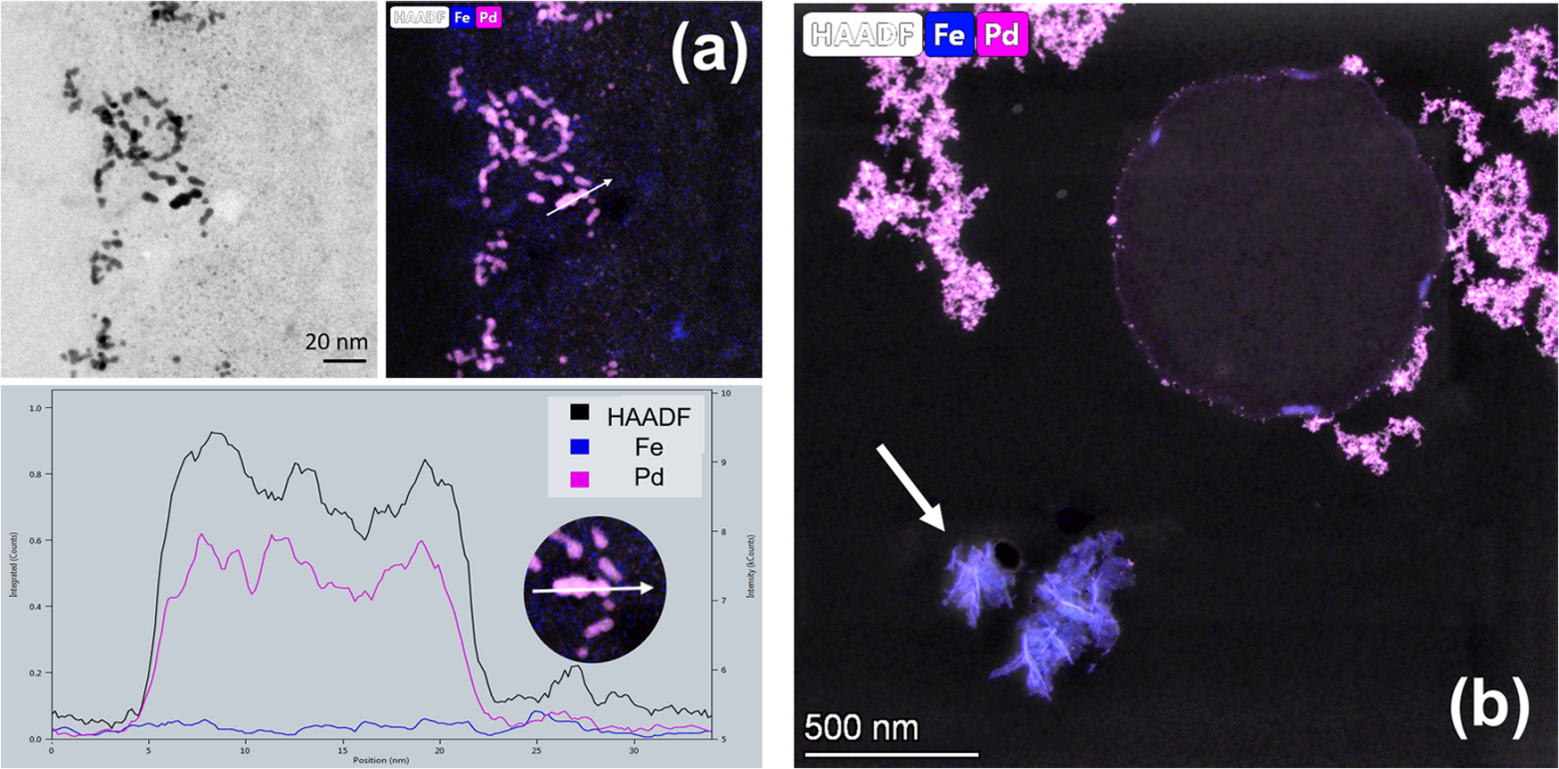
Distribution of Pd and Fe in the samples. (a) EDX cross section of *E. coli*-Pd/Fe_2_ NP clusters on the bacterial cell surface shows that the nanoparticles consist mainly of Pd, with traces of Fe. (b) EM studies indicate the formation of additional structures composed mainly of Fe oxide (white arrow in (b)) in samples exposed to Fe, as seen for *E. coli*-Pd/Fe_1_.

The elemental composition of the specimens was studied using HAADF-STEM with energy-dispersive X-ray spectroscopy (EDX) analysis, revealing the bimetallic nature of the NPs. Dark-field images of Pd and Pd–Fe samples are shown in Figure 6a–d. The image intensity increases with the atomic number; therefore, metals like Pd and Fe appear brighter in the figure compared with the organic material. The EDX mapping shows the colocalization of Pd (pink) and Fe (blue) on the cell surface (Figure 6e–h). Pd was found mainly in the extracellular NP clusters, at the different layers of the cell membrane, and faintly distributed all over the bacterial cytoplasm in all of the specimens. The Fe distribution, on the other hand, is predominantly intracellular with additional large deposits on the cell surface and just a few traces in the extracellular Pd nanoparticles. In the samples exposed to Pd only, the presence of Fe was not detected by the EDX analysis (Figure 6i). The EDX mapping shows that the NPs are richer in Pd than in Fe according to the intensity peaks of each element in the EDX spectra (Supporting Figure S6). The chains of NPs found in the extracellular space and the NPs on both sides of the cytoplasmic membrane were mainly composed of Pd with traces of Fe, as shown in the elemental profile in Figure 7a and Supporting Figure S7. Smaller NPs containing pure Pd were also found, but less frequently, in the *E. coli* intracellular space (Supporting Figure S8). As the concentration of Fe increases, deposits composed mainly of Fe are also found in the samples (indicated by the white arrow in Figure 7b). The Fe deposits may be a result of the reduction of Fe^3+^ ions by the electron-rich medium of sodium formate.

**Figure 8:**
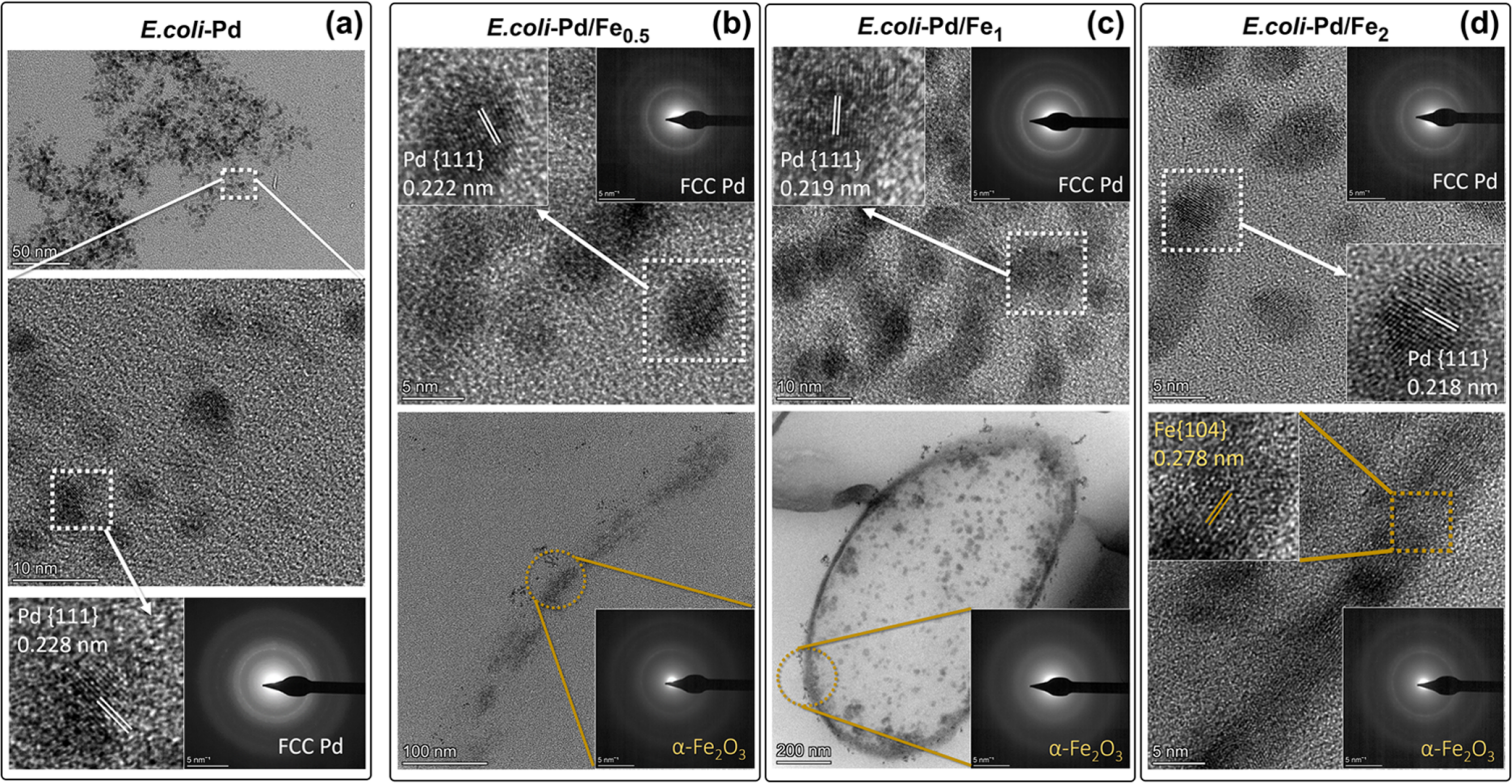
NP Ultrastructure. High-resolution TEM bright field images showing lattice spacing and SAED patterns of biosynthesized (a) extracellular Pd NPs indicate the formation of FCC Pd structure. The lattice spacing and SAED patterns from structures in (b) *E. coli*-Pd/Fe_0.5_, (c) *E. coli*-Pd/Fe_1_, and (d) *E. coli*-Pd/Fe_2_ were found by HR-TEM image analysis. The top and bottom images (b–d) correspond to Pd-rich and Fe-rich regions, respectively, in cells loaded with Pd and Fe.

The HR-TEM study revealed the crystalline morphology of the Pd (Figure 8a) and Pd–Fe (Figure 8b–d, top images) NPs. The small size of the membrane-localized NPs prevented the analysis of their crystal lattice. However, the larger, extracellular NPs showed *d*-spacing of 0.228, 0.222, 0.219, and 0.217 nm for the lattice fringes in the *E. coli*-Pd, *E. coli*-Pd/Fe_0.5_, *E. coli*-Pd/Fe_1_, and *E. coli*-Pd/Fe_2_ samples, respectively, that corresponds to the {111} facet of Pd (insets of Figure 8a–d). The slight decrease in the lattice parameter with the increase in the molar concentration of Fe is consistent with what is reported for Pd–Fe alloys. Pd alloys with unit cells smaller than those in pure Pd, such as Pd–Fe alloys, fall into the category of “contracted” alloys.^70^ The compressive strain in the Pd-based alloys can result in increased orbital overlap, expansion of the *d*-bandwidth, and weakening of adsorption strength.^71^

The diffraction ring patterns on the selected area electron diffraction (SAED) micrographs at Pd-rich regions in the samples were assigned to the crystallographic planes {111}, {200}, {220}, {311}, {222}, {400}, and {331}, which correspond to FCC Pd, based on the reference JCPDS card No. 046–1043 (Supporting Figure S9).^72^

The bottom parts of Figure 8b,c show a view of Fe-rich regions in *E. coli*-Pd/Fe_0.5_, *E. coli*-Pd/Fe_1_, and *E. coli*-Pd/Fe_2_ samples together with their SAED patterns that indicate the presence of a large amount of amorphous Fe. At higher Fe concentrations (bottom part of Figure 8d), it is also possible to observe and measure crystal structures of the iron-rich phase. The crystal structure has a *d*-spacing of 0.27 nm corresponding to the Fe{104} facet of α-Fe_2_O_3_, a nonferromagnetic phase of iron oxide.^73^ Also, the SAED rings match the lattice planes {012}, {110}, {006}, {202}, {122}, {214}, and {306} (Supporting Figure S9) found in a standard for α-Fe_2_O_3_ (hematite) in the American Mineralogist Crystal Structure Database (AMCSD card no. 0000143).^74^

#### Magnetic Properties of the Microbial Pd–Fe NPs

**Figure 9:**
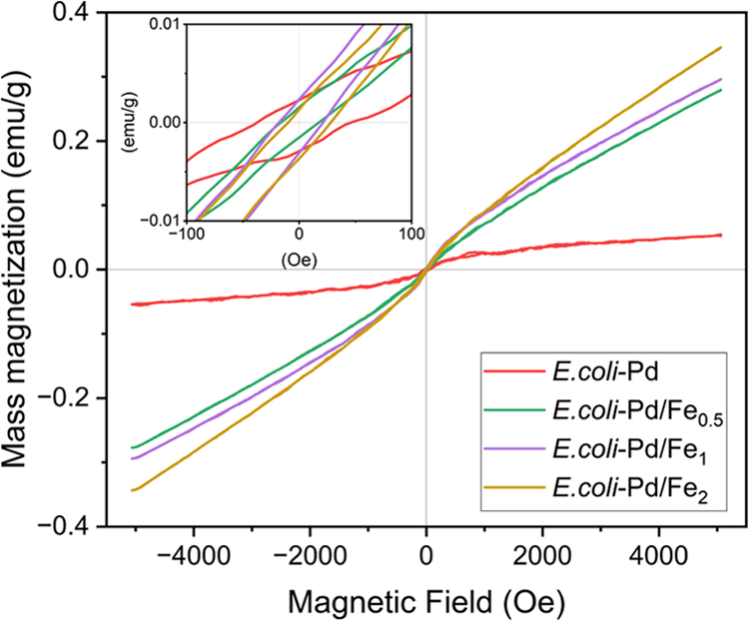
Magnetometry measurements. The magnetic moment data have been normalized to the total mass of metal (Pd + Fe) following background subtraction of the bacterial control sample without metal. The inset shows a closer view of the low field response area, revealing the coercivity and remanent magnetic moment values.

The magnetic properties of the biosynthesized NPs were characterized by vibrating the sample magnetometry (VSM). Figure 9 shows the magnetic moment of the samples in response to a variable magnetic field (−5 to 5 kOe). The amount of Pd and Fe in each sample was quantified by AAS, and the magnetic moment was normalized to the total metal mass (Pd + Fe) in the samples. A background correction was made by subtracting the magnetic moment of the sample holder with *E. coli* bacteria biomass without metal loading. The apparent saturation moment (*M*_s_) of the Pd NPs obtained after subtracting the linear component was equal to 0.05 emu/g at room temperature (∼300 K). Bulk Pd (FCC structure) is anomalously paramagnetic on the verge of being ferromagnetic; however, published works show that Pd NPs in sizes <10 nm can display ferromagnetic properties.^75−78^ The results here suggest a weak magnetic moment related to ferromagnetic or superparamagnetic behavior coming from Pd NPs embedded in the bacterial biomass. Nouh et al.^79^ suggested that the induction of ferromagnetism in Pd NPs can be explained by the interaction between biological capping agents and the NP surface. The surface effects of hydroxyl groups on Pd atoms lead to an increase in the 4d density of states, which accomplishes the Stoner criterion for ferromagnetism.

The maximum magnetic moments per unit of mass (mass magnetization, *M*) measured for Pd–Fe NPs of Pd/Fe ratios 1:0.5, 1:1, and 1:2 were 0.27, 0.29, and 0.34 emu/g, respectively. In all our samples, the initially added Pd concentration is constant, while only Fe content is modified. As expected, the magnetic moment of the NPs increases with the increase of Fe concentration, even when the actual metal uptake in the samples differs slightly from the nominal concentration.^80,81^ The moment rises continuously with the applied magnetic field without any tendency for saturation; this, along with extremely low coercivity values (inset Figure 9), is typical for systems in the superparamagnetic and paramagnetic regime.^82^ The mass magnetic susceptibility of the Pd–Fe NPs produced in this work is weak compared to the values reported earlier for biosynthesized α-Fe_2_O_3_–NPs (1.8 emu/g) and commercial α-Fe_2_O_3_–NPs (0.6 emu/g).^83^ However, the *M*_s_ reported by others was measured as a response to larger magnetic fields (up to 15 kOe). From the increasing mass magnetization values of our samples, it is reasonable to assume that the Pd–Fe NPs produced in this work would possess similar *M*_s_ values in response to similar stronger magnetic fields.

### Catalytic Activity of Pd and Pd–Fe NPs

Pd-based compounds produced by different physicochemical methods are the most common catalytic materials currently used in industrial processes. Biologically produced Pd NPs, as reported by others, offer strong catalytic activity similar to that of commercially available Pd-based catalysts. One example is given in the experiment by Deplanche et al.,^47^ in which the performance of microbially synthesized Pd NPs was comparable to that of a commercially available Pd/C catalyst in the reduction of Cr(VI) to Cr(III).

The model reaction of 4-nitrophenol (4-NP) reduction to 4-aminophenol (4-AP) in the presence of NaBH_4_ is often used to test the catalytic properties of metal NPs. The reduction of 4-NP by NaBH_4_ is not favorable under normal experimental conditions and cannot easily proceed without a catalyst.^29^ However, the notable catalytic properties of Pd NPs can promote this reaction. The reduction of the bright yellow 4-NP compound is easily monitored using time-dependent UV–visible spectra when it is transformed into the colorless 4-AP. The absorption peak is found at a wavelength of 400 nm, which originates from the formation of 4-nitrophenolate ions following the deprotonation of 4-nitrophenol by NaBH_4._^38^ After the addition of the catalyst, this peak diminishes over time, along with the appearance of a new peak at 300 nm, confirming the formation of 4-AP. In this work, microbial production of Pd NPs supplemented with Fe was carried out, presuming that the synergy between Pd and Fe would result in NPs with improved properties for use in catalytic reactions. The freeze-dried samples containing biosynthetic Pd–Fe NPs with different Pd/Fe ratios were tested using 4-NP reduction to 4-AP and compared with the catalytic activity of the Pd-only NPs.

The UV–visible spectra of 4-NP solution exposed to *E. coli*-Pd, *E. coli*-Pd/Fe_0.5_, *E. coli*-Pd/Fe_1_, and *E. coli*-Pd/Fe_2_ samples as a function of time used to monitor 4-NP reduction to 4-AP are shown in Supporting Figure S10. After 30 min, the reaction is almost complete for all catalysts. The possible interference of biological material and added Fe in the reaction was ruled out using the control samples, also shown in Supporting Figure S10. As expected, the solution of 4-NP in the presence of NaBH_4_ alone presented no reduction in time (Supporting Figure S10(a)). A similar result was observed after the addition of the *E. coli* and *E. coli*-Fe samples, as no 4-NP hydrogenation was observed (Supporting Figure S10 (b,d)). In contrast, heat-killed cells loaded with Pd displayed some catalytic activity that was inferior to the Pd and Pd–Fe NPs samples but high enough to confirm the adsorption and reduction of a small amount of Pd in the dead cells (Supporting Figure S10(c)).

**Figure 10:**
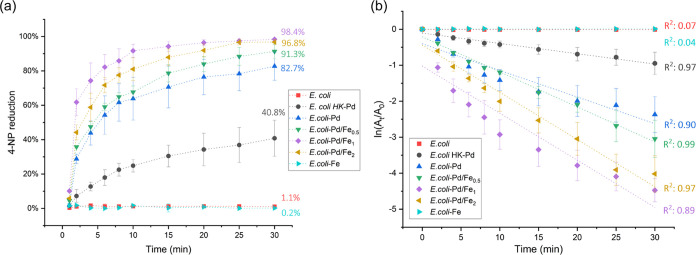
Catalytic activity of the samples. (a) Spectrometric measurement of catalytic activity and (b) fitting of a linear pseudo-first-order kinetic model for the reduction of 4-nitrophenol (4-NP).

The catalytic performance for all of the samples is compared in Figure 10a. After 30 min of reaction, the control samples, *E. coli* and *E. coli*-Fe, reduced only about 1.0% of 4-NP to PAP. Therefore, the bacterial cell components on their own and the *E. coli*-Fe sample have no substantial catalytic activity for this reaction. The *E. coli*-Pd/Fe_1_ sample had the best performance for the reaction with a 4-NP reduction of 98.4% after 30 min, followed closely by *E. coli*-Pd/Fe_2_ with 96.8%. In general, samples with both Pd and Fe are more effective catalysts, in comparison with pure Pd and Fe NPs for the reduction of 4-NP.

Under an excess of NaBH_4_, it is possible to assume that the reaction rate is almost independent of the NaBH_4_ concentration and can be described by a pseudo-first-order reaction eq (eq 1)
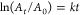
1where *A*_0_ and *A*_*t*_ are the concentrations of 4-NP at time 0 and *t*, respectively. The apparent reaction rate is then the constant *k*, which is obtained from the slope of the linear function when plotting ln(*A*_*t*_/*A*_0_) vs *t*. A second-order kinetic model was also evaluated for this catalytic reaction (Supporting Table S1); however, the average correlation coefficient (*R*^2^) was higher for the pseudo-first-order model across all samples. This suggests that the reaction dynamics follow a pseudo-first-order mechanism, where the rate is directly proportional to the concentration of a single reactant, and this model was selected for further analysis. Figure 10b shows the linear regression (and corresponding *R*^2^ values) of the experimental data used to estimate the apparent reaction rate (*k*) of each sample. The control samples, namely, *E. coli* biomass and *E. coli-*Fe, virtually have no measurable catalytic activity and therefore no slope. The samples *E. coli*-Pd/Fe_1_ and *E. coli*-Pd/Fe_2_ demonstrated similar reaction rates of 0.1308 ± 0.0168 and 0.1312 ± 0.0081 min^–1^, respectively, almost twice higher than the *k* value found for biosynthesized monometallic Pd NPs (see Table 3). Therefore, NPs made of Pd–Fe are more effective in comparison with pure Pd and Fe NPs for the reduction of 4-NP. It is possible to see an increase of the reaction rates proportional to the molar concentration of Fe in the samples. However, the similarity between the reaction rates of *E. coli*-Pd/Fe_2_, which has the highest molar concentration of Fe, and *E. coli*-Pd/Fe_1_ indicates that saturation is reached at the highest Fe concentration. At higher amounts of Fe, the reaction rates reach a plateau, possibly due to a limitation in the incorporation of Fe into the NPs or, alternatively, due to the increase in particle size, which reduces the surface available for interaction with the solution. The observed saturation could also be the result of particle aggregation and the blockage of available active sites in Pd by the crystals of Fe (see TEM results above). In spite of this trend, the distribution of Fe in the NPs produced by this technique is not well defined; therefore, the catalytic activity of each type of NP is difficult to predict.^33^

**Table 3:** Catalytic Reaction Parameters for 4-Nitrophenol (4-NP) Reduction by the Samples at Room Temperature^a^

	Pd concentration (mg L^–1^)	*k* (min^–1^)	*K*_Pd_ (min^–1^ mg^–1^)	TOF (min^–1^)
*E. coli* HK-Pd	0.788 ± 0.041	0.029 ± 0.002	18.20 ± 2.06	0.310 ± 0.035
*E. coli*-Pd	1.793 ± 0.183	0.074 ± 0.009	20.62 ± 4.53	0.351 ± 0.077
*E. coli*-Pd/Fe_0.5_	1.916 ± 0.003	0.097 ± 0.003	25.40 ± 0.92	0.433 ± 0.016
*E. coli*-Pd/Fe_1_	1.935 ± 0.007	0.131 ± 0.017	33.80 ± 4.48	0.575 ± 0.076
*E. coli*-Pd/Fe_2_	1.913 ± 0.020	0.131 ± 0.008	34.29 ± 2.48	0.584 ± 0.042

a Parameters were calculated from the data in Figure 10.

To consider the effect of the variation in metal loading in the samples, to properly assess NPs effectiveness, and to obtain a quantitative comparison with other works, the apparent catalysis rate (*k*) is normalized by the amount of active metal. As previously found, samples containing only Fe have no evident catalytic activity in this reaction. Subsequently, we assume that the different catalytic efficiency in the samples comes from modifications in Pd properties due to the interaction with Fe rather than from the addition of Fe itself; therefore, only Pd is considered as an active metal. The normalized catalysis efficiency (*K*_Pd_) is calculated as *K*_Pd_ = *k*/*M*_Pd_, where *M*_Pd_ is the Pd mass (mg). This helps to identify the nanomaterial that provides the highest catalytic activity per unit weight of metal (Pd). The intrinsic efficiency of the metal, also known as turnover frequency (TOF), is defined as the initial number of 4-NP moles (*n*_4-NP_) reduced by moles of Pd (*n*_Pd_) per minute,  (see Table 3). Notably, after taking the variation in the actual metal content into account, the activity parameters still indicate better performance in the catalytic reduction of 4-NP to 4-AP of the biosynthesized bimetallic Pd–Fe NPs compared to the monometallic Pd NPs.

It should be noted that the heat-killed *E. coli* samples loaded with Pd also showed significant catalytic activity comparable to that in the sample with pure Pd NPs after metal weight normalization. Similar results have been described before by Deplanche et al.^47^ This can be explained by the effect of Pd being passively adsorbed on the bacterial biomass. Although no extracellular or intracellular NPs were visualized in the TEM studies (see above and Supporting Figure S4(a)), just like in the sample with monometallic bio Pd NPs, it is possible that small amounts of Pd are adsorbed in these control samples. After chemical reduction, the scattered Pd clusters form very small NPs that favor interaction with the reactants due to the large available surface area and that are hard to resolve with the available methods.^47^ As the NP size seems to be an important factor for the catalytic features of the samples, future work on microbial synthesized NPs may involve the purification and size separation of the extracellular and intracellular NPs to test differences in their catalytic activity.

The enhanced performance of composite Pd–Fe materials for 4-NP reduction is in agreement with what has been reported in previous studies.^67,84−86^ Due to the electronic structure of Pd, the interaction with other elements, among them Fe, promotes remarkable changes in the electronic density.^86^ The Pd–Fe alloying or even just close contact between these two metals results in electron transfer from Pd to Fe or iron oxide.^87,88^ The displacement of electrons increases the concentration of holes in the Pd d-band, facilitating hydrogenation, as they can now be filled with electrons from the absorbed hydrogen, as well as improves activation of 4-NP when it comes in contact with the Pd NPs surface.^29,84,87^ The strong affinity of Pd to H and O-species can slow down the breaking and formation of new bonds during the catalytic reactions; the compressive strain in Pd–Fe contracted alloys weakens this affinity and thus improves the catalytic performance if the strain is not excessively strong.^71^ Then, smaller unit cell alloys, such as Pd–Fe, would absorb and release hydrogen more efficiently. This effect is also described in other published works (see Table 4) on Pd–Fe-based nanomaterials that demonstrate reaction rates *k* as large as 1.47 min^–1^ (Pd–Fe/G@NC),^89^ 0.328 min^–1^ (PdAu/Fe_3_O_4_),^34^ and 0.0068 min^–1^ (Fe_3_O_4_@Triazole-CS@NNN-Pd),^85^ which are comparable with the ones reported in this work.

**Table 4:** Catalytic Efficiency Comparison between *E. coli*-Pd/Fe_1_ and Other Pd-Based Catalysts toward the 4-NP Reduction Reaction

catalyst	catalyst concen (mg/mL)	NaBH_4_ concen. (mM)	4-NP concen. (mM)	rate constant (*k*) (min^–1^)	mass-normalized rate constant (*K*_pd_) (min^–1^ mg^–1^)	TOF (min^–1^)
biologically synthesized						
Pd–Pt NPs (*S. oneidensis*)^29^	0.99 (mM)	422	1.43	0.0316	7.86	NR
Pd NPs (*S. oneidensis*)^90^	0.025	4.6	0.04	0.0222	15.6	0.59
Pd NPs (cork extract)^40^	0.1	250	0.025	1.248	NR	NR
Pd NPs (eggshell membrane)^40^	0.3	40	0.2	0.135	NR	3.3 × 10^–5^ (mmol mg^–1^ min^–1^)
PdAu/Fe_3_O_4_ (*S. oneidensis*)^34^	0.001	21.1	0.7	0.328	0.2904 (L min^–1^ mg^–1^)	NR
Pd–Fe NPs (*E. coli*)^a^	0.01	20	0.1	0.131	33.8	0.575
chemical/physical synthesized						
Pd–Fe/G@NC^89^	0.1	2000	20	1.47	1.67	613.2
Pd/G@NC^89^	1	1000	10	NR	NR	21.45
Fe_3_O_4_@triazole-CS@NNN-Pd^85^	0.08	4.22	0.08	0.0068	NR	NR
Fe_3_O_4_/graphene/PtPd^91^	2	600	1.85	2.208	NR	76.91
1.37%Pd/O–CNT^92^	0.25	20	1	0.34	NR	3.9
Pd NPs@MOF^93^	0.38	10.9	0.498	NR	NR	0.24

a This work; NR: not reported.

Microbially synthesized Pd-based NPs reported by Tuo et al.^29^ and Xiong et al.^90^ show reaction rates (*k*) for 4-NP hydrogenation of 0.0316 min^–1^ (biogenic Pd–Pt NPs) and 0.0222 min^–1^ (biogenic Pd NPs) (Table 4). Based on the comparison with these biologically produced NPs, our samples appear to be efficient catalysts, at least in the reduction of 4-NP. On the other hand, examples of chemically produced Pd-based materials (Table 4) report TOF values of 21.45 min^–1^ for Pd/G@NC,^89^ 76.91 min^–1^ for Fe_3_O_4_/graphene/PtPd,^91^ and 3.9 min^–1^ for 1.37%Pd/O–CNT.^92^ In these cases, the turnover frequency values are well above the ones measured in this study. Therefore, further improvement of the catalytic efficiency in this reduction reaction is still desirable. The calculated TOF values of the samples with different ratios of Pd and Fe show an increase in the catalytic activity of the nanomaterials with the molar concentration of Fe. From a cost-effectiveness perspective, the modification of the material with inclusions of Fe is ideal for better resource utilization, as it promotes the synthesis of a more efficient catalyst with the use of lower amounts of expensive active metal. Nanoparticles composed of Fe are also of special interest in industry due to their magnetic properties. Magnetism is an attractive feature for catalysis applications, as it facilitates the removal and reuse of expensive metal nanomaterials. As mentioned before, Pd is known for being paramagnetic; however, the microbial Pd NPs produced by *E. coli* indicated a weak ferromagnetic behavior. The magnetic saturation of the microbial Pd NPs found in this work was on the level of 0.05 emu/g. In contrast, other magnetic nanomaterials show higher magnetic saturation, such as 21.52 emu/g for Fe–Pd NPs biosynthesized using bark extract.^67^ While the addition of Fe traces to the samples has the potential to enhance the magnetic properties of the NPs, the current synthesis protocol still requires improvements to be effective for magnetism-based catalyst removal.

### Catalyst Recycling and Reuse

A key performance indicator of catalyst materials is the ability to remove the catalyst from a reaction for recycling and the stability of the catalyst during multiple rounds of reuse. Based on the magnetic properties of the particles, we tested the potential for recycling in a qualitative experiment using a strong, custom-made magnet (Supporting Figure S11). We observed that Pd NPs embedded in the *E. coli* matrix, both with and without incorporated iron, could be magnetically attracted to some extent but that the magnetic strength of the very small particles and/or the strength of the attracting magnet were not sufficient for easy use in magnetic separation processes. Thus, further modifications will be necessary to enhance the magnetic properties of the microbial NPs for recycling during chemical synthesis processes, wastewater treatment, or other potential applications.

**Figure 11:**
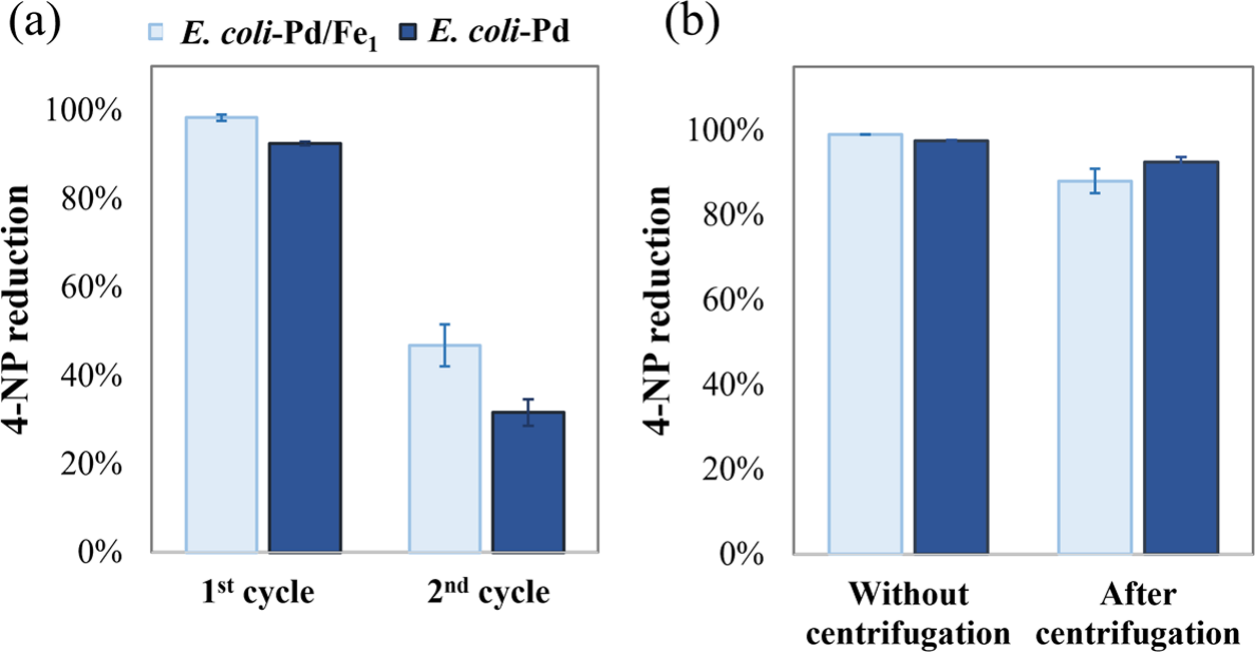
Reusability of *E. coli*-Pd and *E. coli*-Pd/Fe_1_. (a) Both catalyst materials were used in the first reaction and reused for a second cycle of 4-NP reduction. To evaluate potential material loss between cycles, (b) an additional test compared the catalytic activity of the “fresh” catalysts with samples that were centrifuged and resuspended prior to use.

To assess the catalyst reusability, we subjected the material to two rounds of the same reaction, removing the catalyst material by centrifugation in a test tube. Figure 11a shows that both *E. coli*-Pd and *E. coli*-Pd/Fe catalysts achieved high initial catalytic activity, with over 90% reduction of 4-NP in the first cycle. However, a significant drop was observed in the second cycle, with *E. coli*-Pd/Fe and *E. coli*-Pd showing 46.8 and 31.6% reduction, respectively. To assess if this loss of catalytic activity could be attributed to the loss of catalyst material during the centrifugation process, we performed a control experiment (Figure 11b), comparing the activity of a centrifuged sample to a sample where the same initial amount of catalyst was added without prior centrifugation. Both catalyst samples retained >85% activity, suggesting minimal material loss during centrifugation. Thus, the observed activity reduction in the reuse experiment is most probably due to structural or surface changes in the NPs, perhaps from degradation of the biological material that caps the particles (organic molecules). Further studies will be necessary to identify these capping molecules, to clarify the mechanisms behind any activity loss, and to evaluate the catalytic stability under varied reaction conditions. Such studies will guide improvements in catalyst design and stability.

## Conclusions

In this study, Pd–Fe NPs were synthesized using a biological method that relies on live bacteria; it is worth noting that bacteria that were heat-treated prior to incubation with the metal ions did not produce measurable quantities of the NPs. *E. coli* was selected as the model production organism due to its rapid growth and well-studied growth requirements, which make it optimal for industrial applications. *E. coli* also has the potential to be easily genetically engineered to express (or knockout) specific genes related to the reduction of metal ions that may lead to the formation of improved metal NPs. The characterization of our bimetallic Pd–Fe NPs revealed that they are mostly composed of Pd with only traces of Fe; interestingly, they display two differently sized populations depending on their cellular localization: extracellular (2.2 nm) or intracellular (1.3 nm). The interaction of Pd and Fe with P=O, −CH_2_, and −CH_3_ functional groups indicated by the FT-IR studies suggests their interactions with membrane lipids; the involvement of lipids in bacterial metal uptake and the further formation of NPs is not understood and should be investigated further. We demonstrate that the bimetallic Pd–Fe NPs are efficient catalysts in the 4-NP model reduction reaction and that the catalytic activity is greatly enhanced with the addition of Fe compared to pure Pd NPs. Pd–Fe NPs exhibit significantly faster reaction kinetics and higher conversion rates than their monometallic counterparts. The apparent reaction rate increased with an increasing Fe content of the samples. However, saturation is reached at high concentrations of Fe, possibly due to an increase in particle size or a limit on the amount of Fe that can be incorporated into the Pd-based NPs. By the use of Pd–Fe alloys, the amount of precious Pd metal required for catalysis can be reduced, making catalysis with Pd NPs more economically viable. Taken together with a biological synthesis method that allows us to take up and reduce Pd ions from dilute solutions, our system is attractive for a circular economy approach, where Pd catalyst waste can be oxidized and fed back to bacteria to generate new catalyst materials quickly and efficiently.

## Methods

### Bacterial Strain and Culture Conditions

In this study, *E. coli* K-12 BW25113^94^ was used for the production of Pd and Pd–Fe NPs under anaerobic conditions. A single colony of *E. coli* was inoculated in 50 mL Falcon tubes filled with lysogeny broth (LB) and incubated at 37 °C with 200 rpm shaking until the optical density (OD_600_) reached 0.5. Anaerobic conditions were approximated in our experiments by filling the Falcon tubes to the top with the medium, resulting in a minimal oxygen diffusion into the samples.^95^ Cells were harvested by centrifugation at 4200*g* for 10 min, washed two times with 20 mM MOPS buffer (pH 8), and normalized to OD_600_ equal to 3.5, which is equal to 2.5 × 10^9^ CFU/mL or 1.2 ± 0.1 mg dry weight/mL. All of the bacterial samples were used immediately after preparation, as described below.

### Biogenic Synthesis of Pd and Pd–Fe NPs

The NPs were synthesized using a modified method of Pd(II) reduction by bacterial cells described by Courtney et al.^42^ Stock solutions of Pd(II) (20 mM) and Fe(III) (20 mM) were prepared by dissolving sodium tetrachloropalladate(II) trihydrate (Na_2_PdCl_4_·3H_2_O) in 0.01 M HNO_3_ and iron(III) chloride (FeCl_3_) in distilled H_2_O. For monometallic Pd NPs, 52.6 μL of the 20 mM Na_2_PdCl_4_ solution was added per mL of the 3.5 OD_600_ bacteria suspension to reach a final 1 mM concentration of Pd equivalent to 10% w/w (metal/biomass dry weight) metal loading. All of the bimetallic nanoparticles were prepared with the same loading of palladium (10% w/w), while the molar ratio of Fe was varied as follows: 1:0.5, 1:1, and 1:2 (Pd/Fe). The initial metal mass fractions used in each sample (calculated as a percentage of the cells’ dry weight) were 10% w/w Pd:2.5% w/w Fe, 10% w/w Pd:5% w/w Fe, and 10% w/w Pd:10% w/w Fe. The samples’ denotation and metal amounts added are shown in Table 5. The removal of Pd(II) from the solutions without Fe was monitored by spectrophotometry using the tin(II) chloride (SnCl_2_) assay method, as described previously.^47,96^ In brief, timed samples of 150 μL were withdrawn every 30 min and centrifuged (5900*g*, 4 min). In 96-well microplates, 40 μL of the supernatants was added with 3 replicates. A 52 mM SnCl_2_ solution was prepared in 0.5 M HCL. After 2 h, 160 μL of the SnCl_2_ solution was added to each well and incubated for 30 min at 30 °C. The absorbance at 463 nm was determined and compared to a calibration curve made from the original Pd(II) solution prepared in the same way.

**Table 5:** Starting Concentrations of Palladium(II) and Iron(III) Used in the Samples

sample	[Pd] (mM)	[Fe] (mM)
*E.coli*		
*E. coli* HK-Pd	1	
*E. coli*-Pd	1	
*E. coli*-Fe		1
*E. coli*-Pd/Fe_0.5_	1	0.5
*E. coli*-Pd/Fe_1_	1	1
*E. coli*-Pd/Fe_2_	1	2

After 2 h of incubation at 30 °C with agitation at 550 rpm, the cells were washed with 20 mM MOPS buffer (pH 8) to remove the nonabsorbed metal ions, and the reduction was initiated by the introduction of an external electron donor. For this, sodium formate was added to the suspension to make a final concentration of 10 mM. The samples were kept at 30 °C with 550 rpm agitation for approximately 1 h until a black precipitate was observed. The change in color of the solution is frequently used to assess the reduction of Pd(II) to Pd(0).^47^ As a negative control, the same experiment using only a Pd solution was carried out in parallel with heat-killed cells. For heat killing, 2 mL of the bacterial suspension was kept at 80 °C for 15 min, followed by dipping the tube in ice for 2 min. A control sample of *E. coli* cells exposed to Fe only (5% w/w) was also prepared.

### Characterization of Biogenic NPs

#### Fourier Transform Infrared Spectroscopy and X-ray Diffraction

The Pd and Pd–Fe NPs were washed three times with distilled water and concentrated by removing the supernatant after centrifugation at 5900*g* for 2 min. The pellet (bacterial biomass and NPs) was then kept at −80 °C overnight and freeze-dried for 24 h. The FT-IR spectrum of the NP powder was recorded on a PerkinElmer Spectrum 100 FT-IR spectrometer in transmittance mode over the frequency range of 550–4000 cm^–1^. XRD was performed at room temperature on the powdered samples using a Bruker D8 DISCOVER diffractometer at 50 kV and 1 mA with Cu Kα radiation as the X-ray source over the 2θ angle range of 2 to 75°.

#### Thermogravimetric Analysis

The thermal stability of the NPs powder was investigated using TGA. Approximately 10 mg of each biosynthesized Pd and Pd–Fe NPs were characterized with a Discovery TGA 550 analyzer (TA Instruments) in the temperature range from room temperature to 700 °C in a nitrogen atmosphere at a heating rate of 10 °C/min.

#### Atomic Absorption Spectroscopy

The cell pellets with NPs were also analyzed by AAS to quantify Pd(II) and Fe(III) ion uptake by the bacteria. For this technique, the samples were resuspended in 50 μL of a mixture of concentrated nitric acid (65% HNO_3_) and hydrochloric acid (37% HCl) (aqua regia) and left to digest overnight. Afterward, the samples were held for 20 min at 90 °C until the solution became transparent, which is an indication of complete digestion, and further resuspended in 10 mL of HCl 5% (v/v). Measurements were taken using a PerkinElmer AANALYST400 flame atomic absorption spectrometer. The instrument was calibrated using 0.5, 1, 10, 50, and 150 μM dilutions of certified Pd and Fe standard calibration solutions in HCl 5% (v/v). Samples of each kind of nanoparticle were analyzed in triplicate in three independent experiments. Data were combined, and the mean (μ) was calculated as the average of all replicates. The error is expressed as the standard deviation (σ), which is calculated as the square root of the variance, , where *N* is the size of the population and *x*_*i*_ is the value measured for each sample.

#### Scanning Transmission Electron Microscopy with High-Angle Annular Dark-Field Detector and Energy-Dispersive X-ray Spectroscopy

For electron microscopy examination, the NP-loaded cells were washed, fixed, dehydrated, and embedded in resin according to Deplanche et al.^47^ Fixation took place overnight in 4% (v/v) paraformaldehyde and 2% (v/v) glutaraldehyde in 20 mM MOPS buffer (pH 8.0, 4 °C). On the next day, the samples were washed twice with 0.1 M cacodylate buffer and stained with 1% aqueous osmium tetraoxide. Then, dehydration was done using ascending ethanol concentrations of 50, 70, 90, and 96% for 10 min each. Finally, 100% ethanol was used for the dehydration 4 times, for 15 min each. The samples were then incubated in 1 mL of 100% acetone for 15 min, followed by an incubation in 1:1 acetone-EPON resin solution at room temperature overnight. On the following day, the NP-loaded cells were incubated in 100% EPON resin for 1 h at room temperature before finally drying in an oven at 60 °C for 3 days. After the solidification of the resin, a Diatome ultra 45° diamond knife (Diatome Ltd., Switzerland) was used to section the resin into thin slices of 70 nm, which were deposited on 100 μm copper grids. To determine the location, size, and element content of Pd and Pd–Fe NPs in the cells, NP-loaded bacteria were examined in a FEI TITAN G2 80–300 STEM at 300 keV. For elemental analysis of the specimens, EDX was used with a spot size of 4 Å and a live counting time of 50 s coupled with a high-resolution STEM and high-angle annular dark-field (HAADF) detector.

#### Vibrating Sample Magnetometry

The magnetic properties of the samples were investigated by VSM (PMC MicroMag 3900 VSM, Lake Shore Inc., Idea-VSM) at room temperature in an applied magnetic field sweeping from −5 to 5 kOe. A small amount of each dried NP sample (∼0.01 g) was inserted into a gelatin capsule. The material was tightly compacted inside each capsule and then placed inside the cylindrical sample holder in the VSM coil set.

### Catalysis of Hydrogenation of 4-Nitrophenol to 4-Aminophenol

The catalytic activity of *E. coli*-Pd, *E. coli*-Pd/Fe_0.5_, *E. coli*-Pd/Fe_1_, and *E. coli*-Pd/Fe_2_ was studied using the borohydride reduction of 4-nitrophenol (4-NP). The biogenic nanoparticles were freeze-dried as described above and further dispersed in distilled water. For the reduction, 20 mM sodium borohydride (NaBH_4_) and 0.02 g/L NPs were added to 2 mL of 0.1 mM 4-NP. The conversion of 4-NP to 4-aminophenol (4-AP) was monitored with UV–visible light using a spectrophotometer (EasyPlus UV/vis, Mettler toledo) in the wavelength range of 200–500 nm. Three biological replicates of each catalyst type were conducted in independent experiments. The mean and error were calculated as described above. In order to assess the reusability of the catalyst, *E. coli*-Pd and *E. coli*-Pd/Fe_1_ samples were removed from the solution after 4-NP reduction by centrifugation at 17,000*g* for 10 min and resuspended before being reused in a subsequent reaction. All catalyst materials were tested in triplicate.

### Image Processing and Statistical Analysis

To determine the average size of Pd and Pd–Fe NPs produced in different experiments from HR-TEM images as well as their distribution by means, image processing software ImageJ (National Institutes of Health, Maryland, United States) was used. The bandpass filter function was applied to distinguish the NPs in cells from background signals and artifacts, as was previously described in ref (41). Nanoparticles located outside the bacterial membrane were classified as extracellular, while those found in the cytosol or within any of the membrane lipid layers were categorized as intracellular. Each particle size was manually measured by using ImageJ software. Mean particle size was calculated from at least three different areas of samples with more than 200 NPs in each area. Particle size distribution was estimated using Origin 2022b software. Statistical significance between groups was determined using one-way ANOVA, followed by Tukey–Kramer post hoc multiple comparison tests.

## Supporting Information Available

Quantification of metal ion uptake data; X-ray diffraction compared with standard for α-Fe_2_O_3_; TGA and DTG curves of the samples; HAADF-STEM images of control samples; additional HAADF-STEM (inverted contrast) images of the samples; EDX spectra analysis of the NPs at the cell surface; EDX of a cross section of an NP cluster in an *E. coli*-Pd/Fe1 sample; HAADF-STEM images of intracellular nanoparticles; SAED measurements; UV–vis spectra of catalyst experiments with 4-nitrophenol; magnetic separation experiment; and kinetic model constants and correlation coefficients (PDF)

## Abbreviations

4-NP: 4-nitrophenol
4-AP: 4-aminophenol
AAS: atomic absorption spectroscopy
CFU: colony forming unit
DTG: differential thermogravimetry
EDX: energy-dispersive X-ray spectroscopy
FCC: face-centered cubic
FT-IR: Fourier transform infrared spectrometry
HAADF: high-angle annular dark-field
HR-TEM: high-resolution transmission electron microscopy
NPs: nanoparticles
OD: optical density
SAED: selected area diffraction
SQUID: superconducting quantum interference device
STEM: scanning transmission electron microscopy
TEM: transmission electron microscopy
TGA: thermal gravimetric analysis
TOF: turnover Frequency
VSM: vibrating sample magnetometry
XRD: X-ray diffractometry
