# Supplementary material for: Characterization of Bimetallic Pd–Fe Nanoparticles Synthesized in Escherichia coli
Source: ACS Appl Bio Mater. 2024 Dec 2;7(12):8573–89. doi: 10.1021/acsabm.4c01354 (PMC11653405; doi:10.1021/acsabm.4c01354)
Supplement: Supplementary file 1 — mt4c01354_si_001.pdf [file mt4c01354_si_001.pdf]

## Supporting Information

# Characterization of bimetallic Pd-Fe nanoparticles synthesized in *Escherichia coli.*

Ana Lucía Campaña Perilla[1,2], Jaime Gomez-Bolivar[3], Mohamed L. Merroun[3], Nadeem Joudeh[1], Athanasios Saragliadis[1], Anja Røyne[2], Dirk Linke[1]*, and Pavlo Mikheenko[2]*

[1] Department of Biosciences, University of Oslo, P.O. Box 1066 Blindern, 0316 Oslo, Norway
[2] Department of Physics, University of Oslo, P.O. Box 1048 Blindern, 0316 Oslo, Norway
[3] Department of Microbiology, University of Granada, Campus Fuentenueva, 18071 Granada, Spain

*Corresponding authors: dirk.linke@ibv.uio.no and pavlo.mikheenko@fys.uio.no

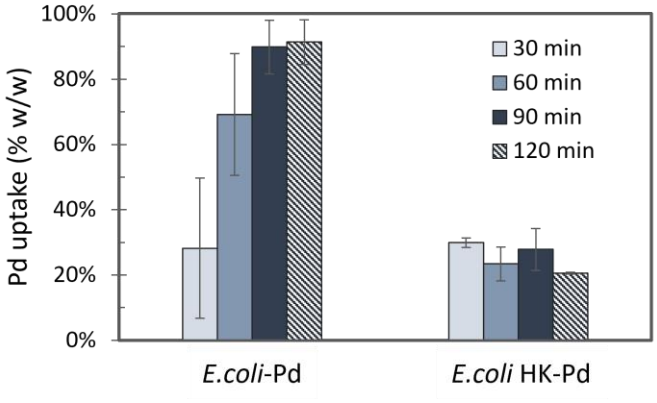


*Figure S1. Quantification of metal ion uptake. Spectrophotometric tin (II) chloride-based measurement of Palladium ion uptake from solution by E. coli and heat-killed E. coli cells at incubation times 30, 60, 90 and 120 min.*

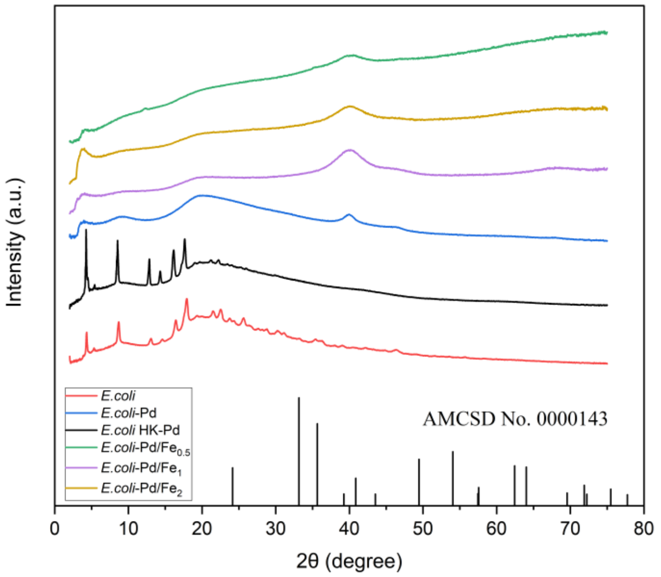


*Figure S2. X-ray diffraction. XRD patterns of the samples are compared to the standard for α-$Fe_2O_3$ (Hematite) in the American Mineralogist Crystal Structure Database (AMCSD card no. 0000143)*

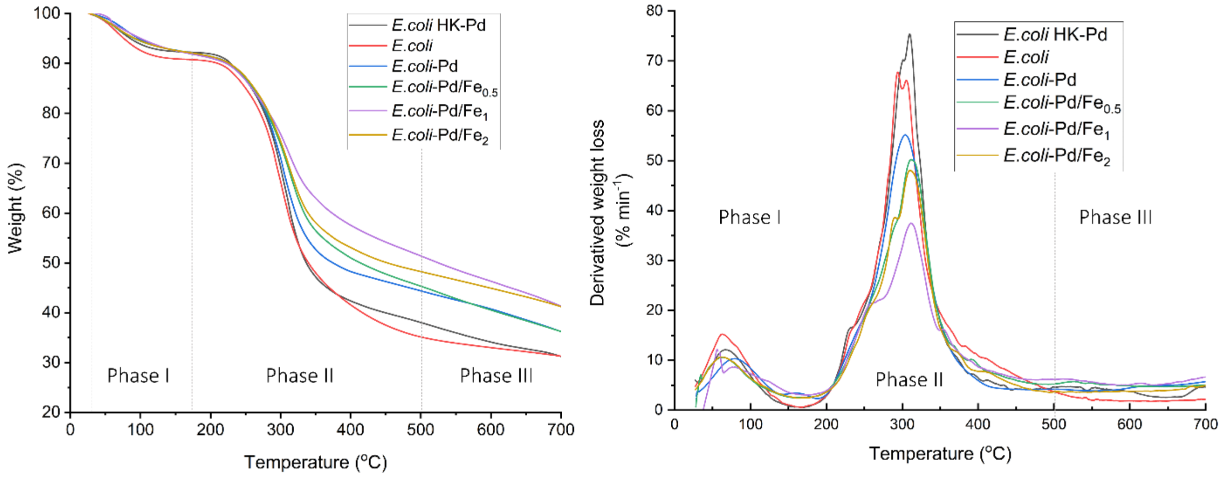


*Figure S3. Thermogravimetric analysis. TGA (weight loss, left panel) and DTG (derivative of weight loss, right panel) of the samples care shown.*

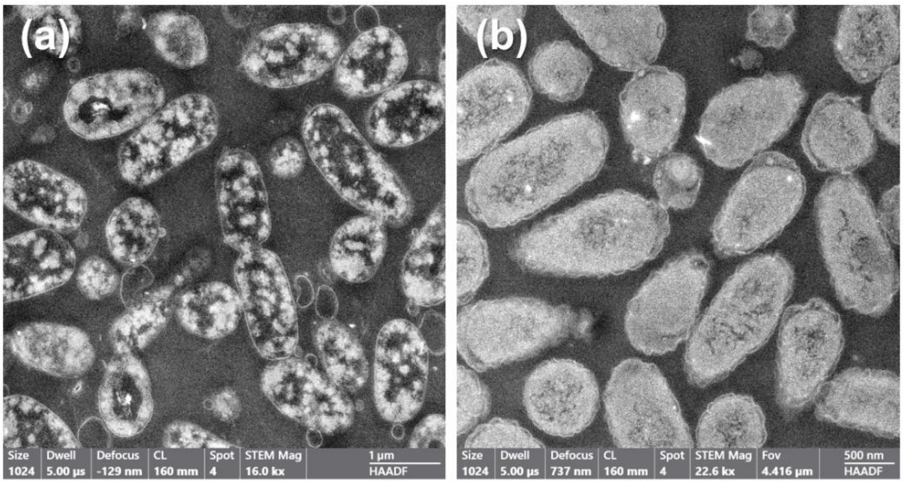


*Figure S4. HAADF-STEM images of control samples. (a) heat-killed E.coli K-12 cells loaded with Pd and (b) unloaded cells.*

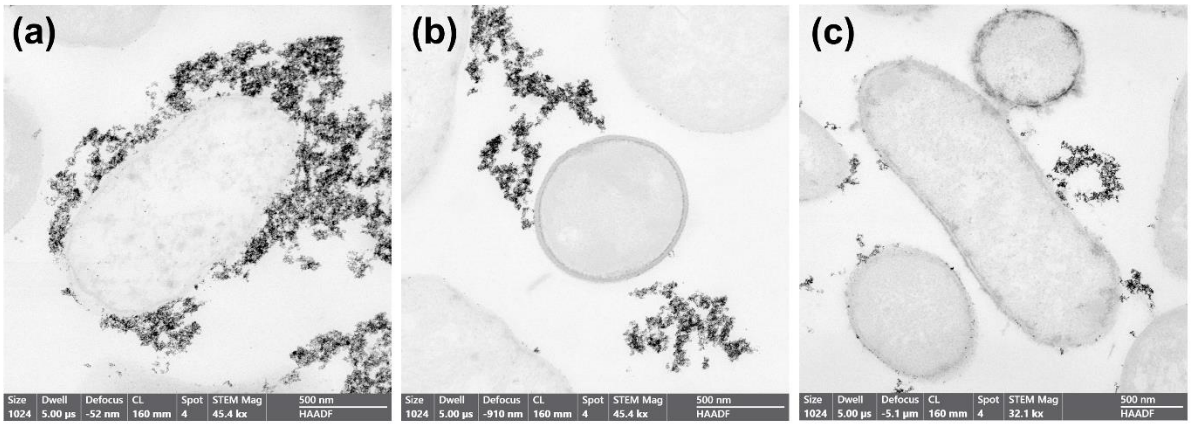


*Figure S5. HAADF-STEM (inverted contrast) images of (a) E.coli-Pd, (b) E.coli-Pd/Fe$_{0.5}$, and (c) E.coli-Pd/Fe$_2$*

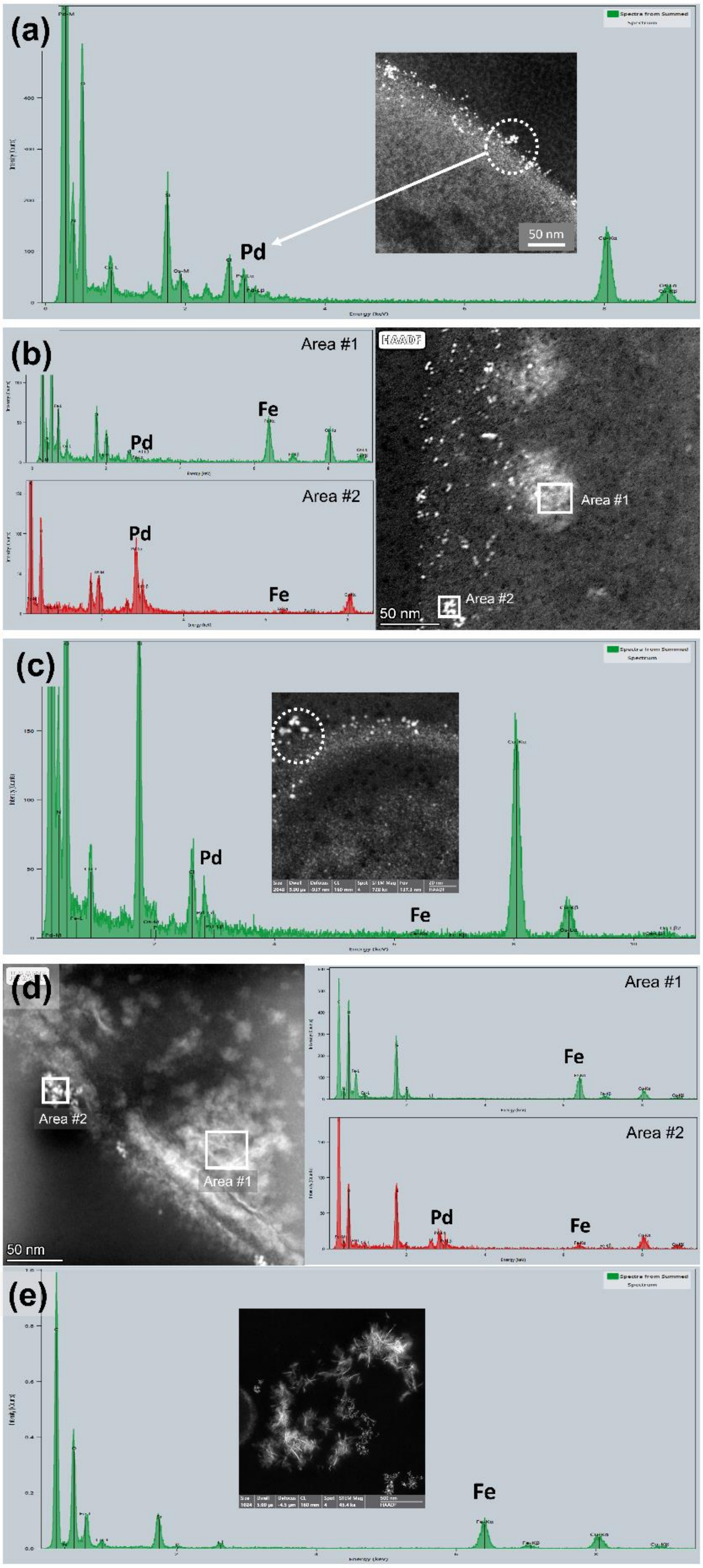


*Figure S6. EDX spectra analysis of the NPs at the cell surface. (a) E.coli-Pd, (b)E.coli-Pd/$Fe_{0.5}$, (c)E.coli-Pd/$Fe_1$, (d)E.coli-Pd/$Fe_2$, and (e) extracellular crystals, made mainly of Fe, in the sample E.coli-Pd/$Fe_1$ shown.*

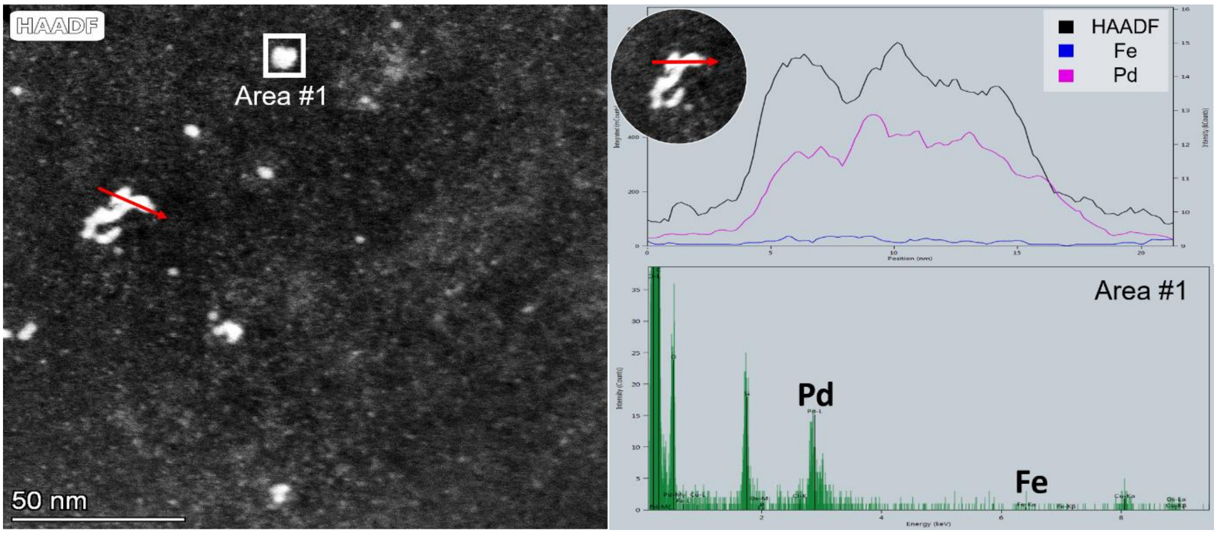


*Figure S7. EDX of a cross-section of an NP cluster in an E.coli-Pd/Fe$_1$ sample. Pd and Fe are co-localized. Traces of Fe were detected with no evidence of homogeneous structures.*

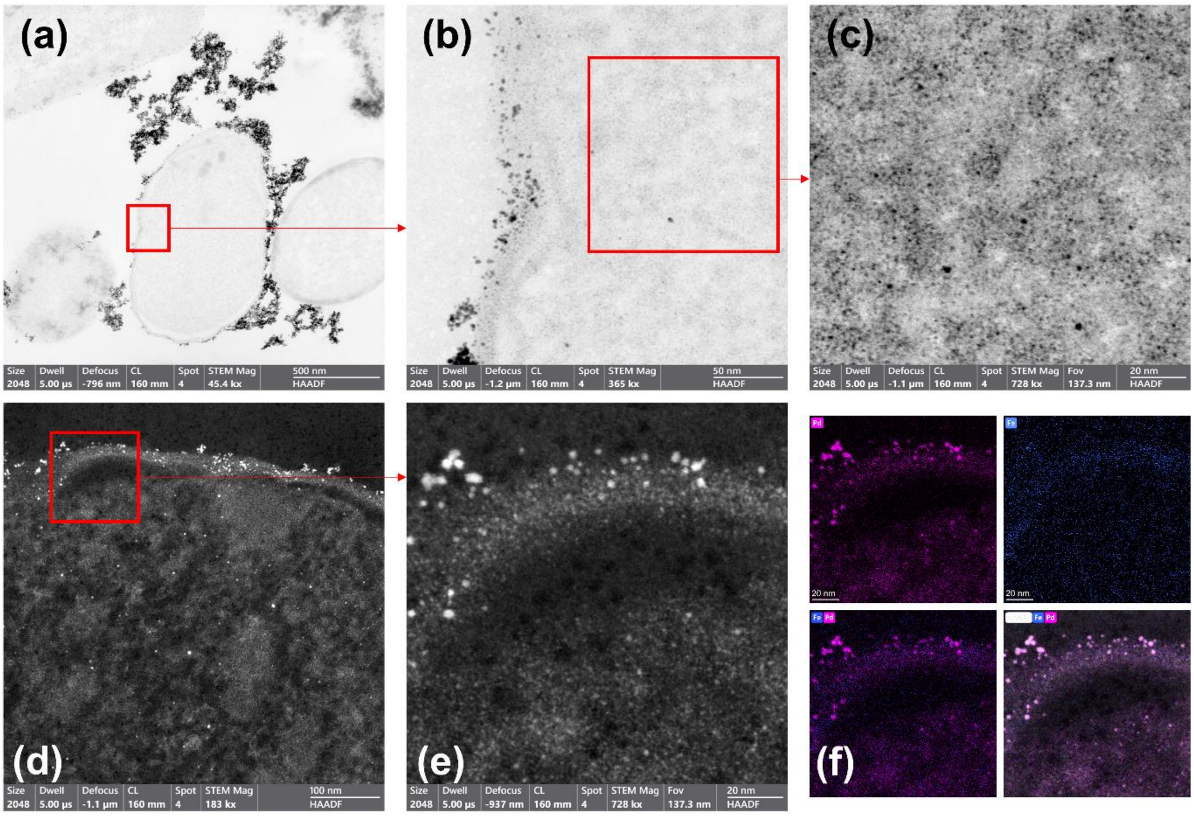


*Figure S8. HAADF-STEM images of intracellular nanoparticles. Inverted contrast and normal dark field images of E.coli-Pd/Fe$_1$ NPs (a,d) and their respective magnification close to membranes (b, e). (c) Further magnification (Inverted contrast) of an area of the bacteria cytosol with dark spots associated with Pd NP formation. (f) EDX distribution map for the membrane region and for intracellular NPs. The small intracellular NPs are composed mainly of Pd while Fe accumulates at the membrane region.*

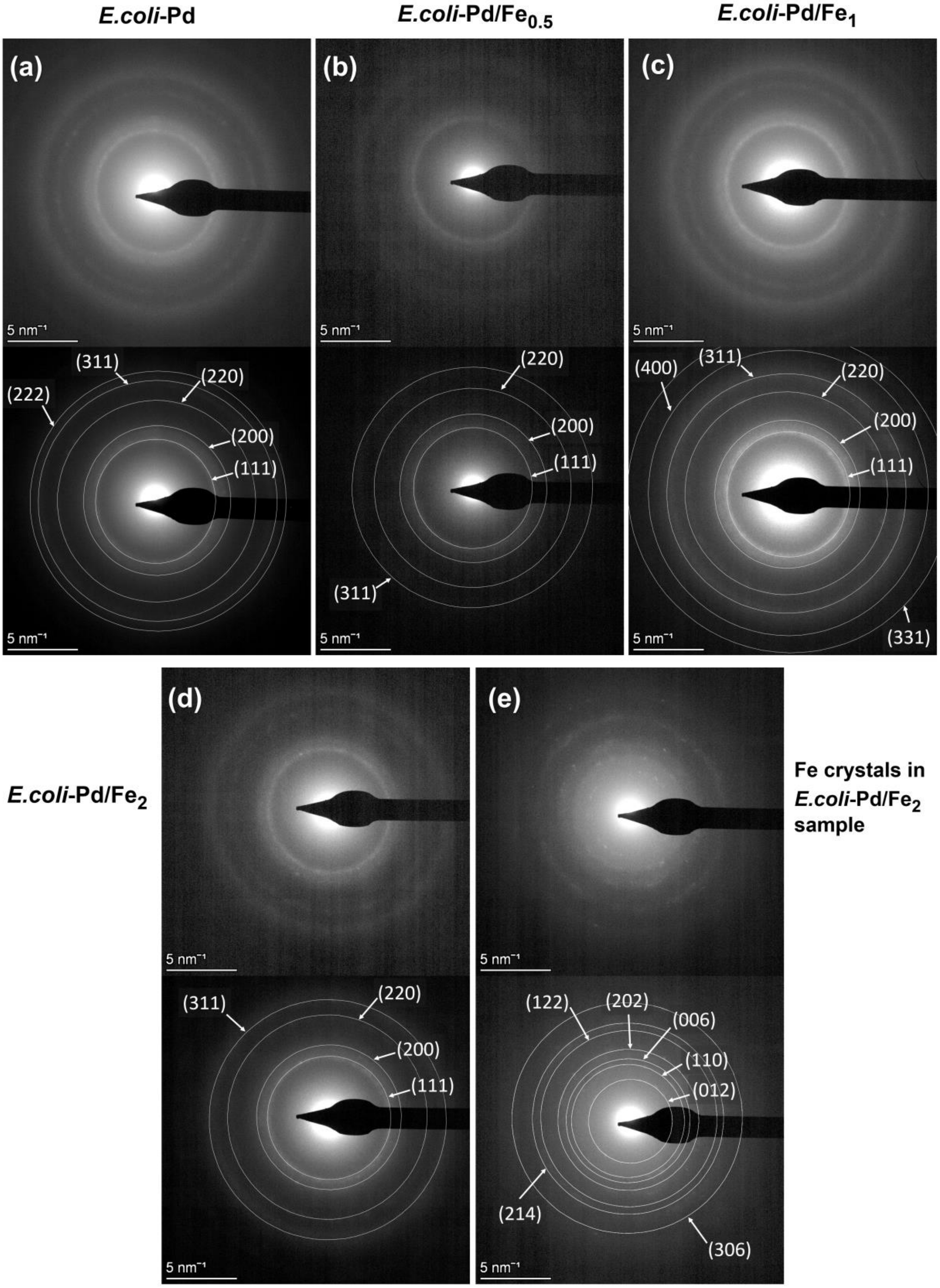


*Figure S9. SAED measurements. Miller index designation with d-spacing measured from rings in SAED patterns of the samples*

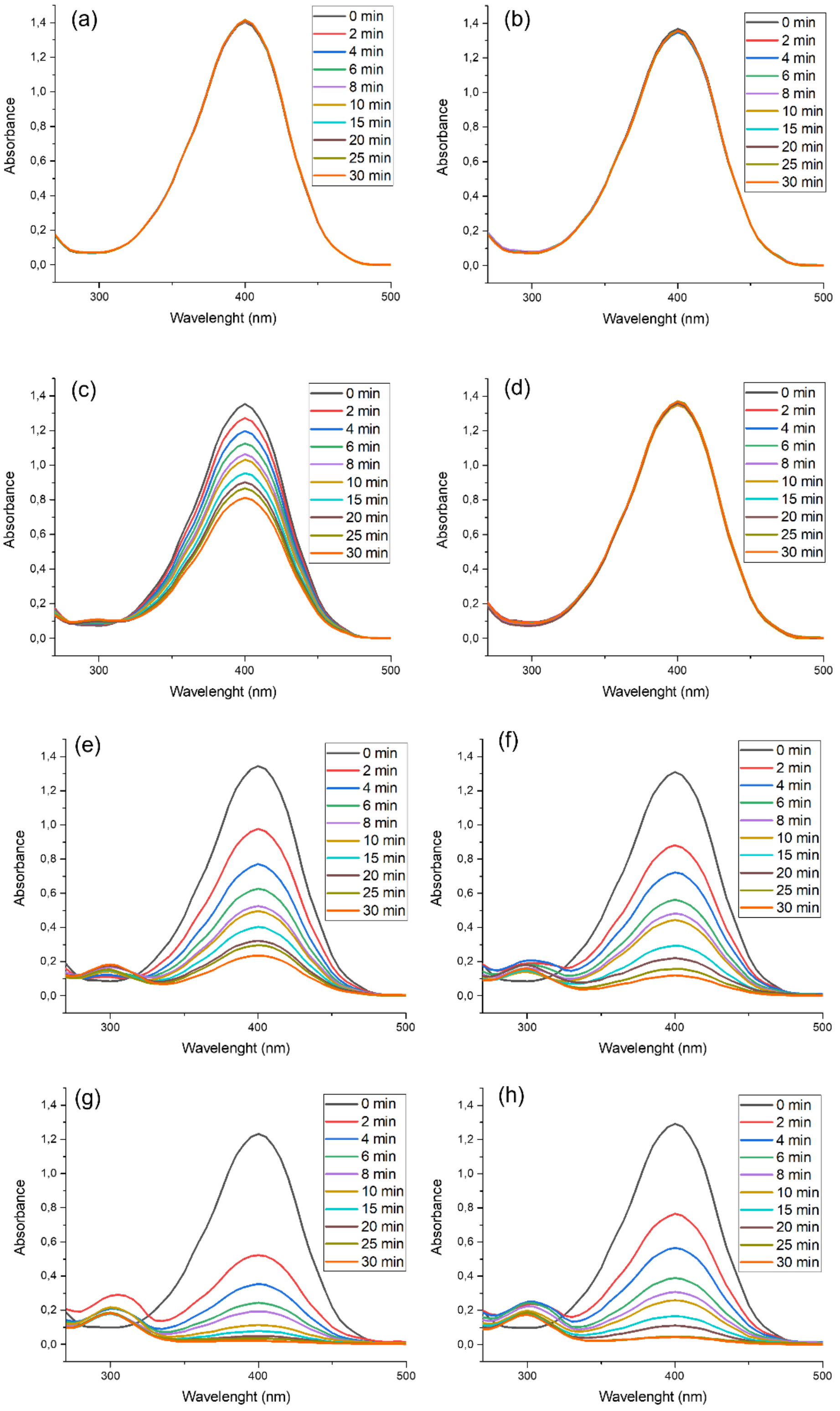


*Figure S10. UV-Vis spectra of catalyst experiments with 4-nitrophenol. UV-visible absorption spectra of 4-nitrophenol in $NaBH_4$ (a) during 30 min incubation at room temperature and 4-nitrophenol reduction to 4-AP by $NaBH_4$ catalysed by the following samples (b) E. coli, (c) E. coli HK-Pd, (d) E. coli-Fe,(e) E.coli-Pd, (f) E.coli-Pd/$Fe_{0.5}$, (g) E.coli-Pd/$Fe_1$, and (h) E.coli-Pd/$Fe_2$*

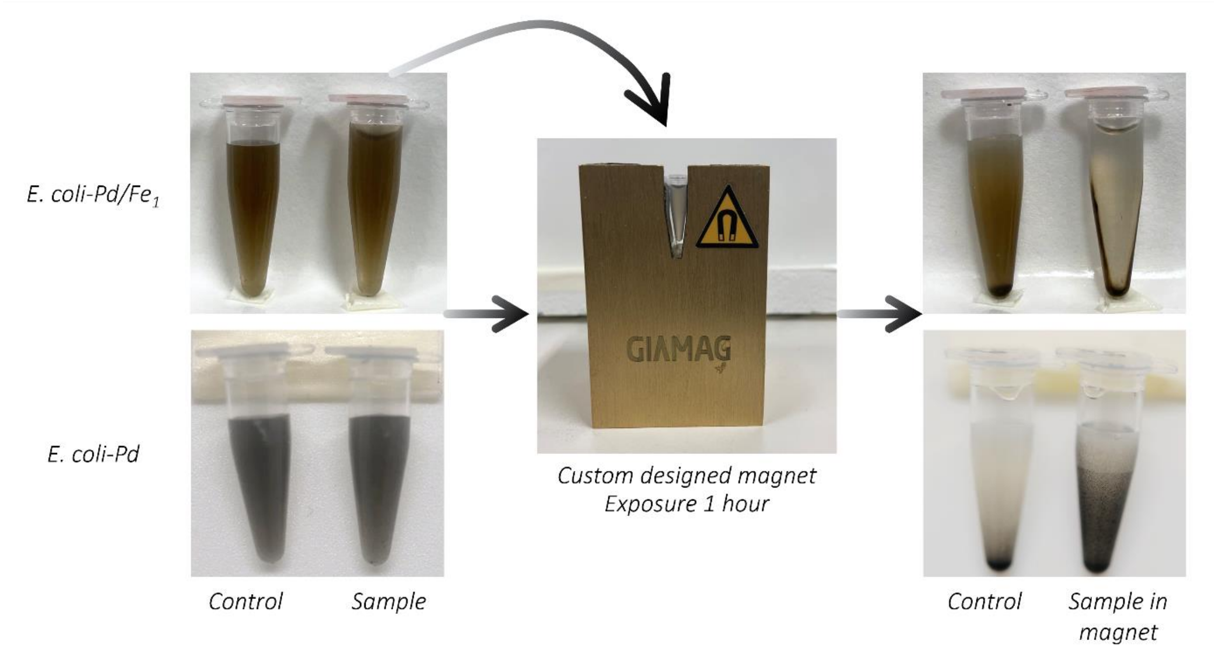


*Figure S11. Magnetic separation experiment. E.coli-Pd and E.coli-Pd/Fe$_1$ NPs samples were exposed to the magnetic field of a custom designed NdFeB magnet of grade N52 (GIAMAG AS, Norway) for 1 hour. The comparation with control samples not exposed to the magnetic field shows the collection of the material at the walls of the Eppendorf tubes where the magnetic field is strongest. The effect is more pronounced in the samples that contain Fe.*

*Table S1. Pseudo-first and second order kinetic model constants and correlation coefficients for the 4-nitrophenol reduction to 4-AP by NaBH$_4$ catalysed by the samples. $A_0$ and $A_t$ are the concentrations of 4-NP at time 0 and t while $k_1$ ($min^{-1}$) and $k_2$ ($M^{-1}min^{-1}$) are the reaction constants for each kinetic model.*

| **Sample** | **Pseudo-first order ($\ln(A_t/A_0)$ vs $t$)** | | | **Second order ($1/A_t$ vs $t$)** | | |
|---|---|---|---|---|---|---|
| | **Intercept** | **Slope (-$k_1$)** | **$R^2$** | **Intercept** | **Slope ($k_2$)** | **$R^2$** |
| ***E.coli*-Pd** | -0.26 | -0.09 | 0.95 | -3.32 | 1.05 | 0.93 |
| ***E.coli* HK-Pd** | -0.07 | -0.02 | 0.96 | 1.03 | 0.04 | 0.98 |
| ***E.coli*-Pd/Fe$_{0.5}$** | -0.12 | -0.12 | 0.96 | -10.47 | 2.24 | 0.81 |
| ***E.coli*-Pd/Fe$_1$** | -0.82 | -0.13 | 0.89 | -10.04 | 3.64 | 0.91 |
| ***E.coli*-Pd/Fe$_2$** | -0.22 | -0.13 | 0.99 | -27.38 | 5.18 | 0.81 |
| **Average $R^2$** | | | **0.95** | | | **0.89** |
